# CXCR7 Controls Competition for Recruitment of β-Arrestin 2 in Cells Expressing Both CXCR4 and CXCR7

**DOI:** 10.1371/journal.pone.0098328

**Published:** 2014-06-04

**Authors:** Nathaniel L. Coggins, Danielle Trakimas, S. Laura Chang, Anna Ehrlich, Paramita Ray, Kathryn E. Luker, Jennifer J. Linderman, Gary D. Luker

**Affiliations:** 1 Center for Molecular Imaging, Department of Radiology, Department of Microbiology and Immunology, University of Michigan, Ann Arbor, Michigan, United States of America; 2 Department of Chemical Engineering, Department of Microbiology and Immunology, University of Michigan, Ann Arbor, Michigan, United States of America; 3 Department of Biomedical Engineering, Department of Microbiology and Immunology, University of Michigan, Ann Arbor, Michigan, United States of America; 4 Department of Microbiology and Immunology, University of Michigan, Ann Arbor, Michigan, United States of America; University of North Dakota, United States of America

## Abstract

Chemokine CXCL12 promotes growth and metastasis of more than 20 different human cancers, as well as pathogenesis of other common diseases. CXCL12 binds two different receptors, CXCR4 and CXCR7, both of which recruit and signal through the cytosolic adapter protein β-arrestin 2. Differences in CXCL12-dependent recruitment of β-arrestin 2 in cells expressing one or both receptors remain poorly defined. To quantitatively investigate parameters controlling association of β-arrestin 2 with CXCR4 or CXCR7 in cells co-expressing both receptors, we used a systems biology approach combining real-time, multi-spectral luciferase complementation imaging with computational modeling. Cells expressing only CXCR4 maintain low basal association with β-arrestin 2, and CXCL12 induces a rapid, transient increase in this interaction. In contrast, cells expressing only CXCR7 have higher basal association with β-arrestin 2 and exhibit more gradual, prolonged recruitment of β-arrestin 2 in response to CXCL12. We developed and fit a data-driven computational model for association of either CXCR4 or CXCR7 with β-arrestin 2 in cells expressing only one type of receptor. We then experimentally validated model predictions that co-expression of CXCR4 and CXCR7 on the same cell substantially decreases both the magnitude and duration of CXCL12-regulated recruitment of β-arrestin 2 to CXCR4. Co-expression of both receptors on the same cell only minimally alters recruitment of β-arrestin 2 to CXCR7. *In silico* experiments also identified β-arrestin 2 as a limiting factor in cells expressing both receptors, establishing that CXCR7 wins the “competition” with CXCR4 for CXCL12 and recruitment of β-arrestin 2. These results reveal how competition for β-arrestin 2 controls integrated responses to CXCL12 in cells expressing both CXCR4 and CXCR7. These results advance understanding of normal and pathologic functions of CXCL12, which is critical for developing effective strategies to target these pathways therapeutically.

## Introduction

Chemokine CXCL12 activates multiple intracellular networks, including mitogen activated protein kinases (MAPK), PI3 kinase-AKT, and JAK-Stat, to control proliferation, survival, chemotaxis, transcription, and other cellular responses [1]–[3]. The numerous signaling pathways regulated by this chemokine correspond with critical functions in development, normal physiology, and disease. Germline deletion of CXCL12 in mice is lethal due to abnormal development of cardiovascular, hematopoietic, and central nervous systems [4]–[6]. CXCL12 controls trafficking of immune cells and homing and retention of hematopoietic stem cells in bone marrow. CXCL12-dependent pathways promote growth and metastasis of more than 20 different human malignancies, and this chemokine also affects pathogenesis of other common diseases such as atherosclerosis, multiple sclerosis, rheumatoid arthritis and diabetes [7], [8].

CXCL12 signals through chemokine receptors CXCR4 and CXCR7 (recently renamed ACKR3). In cells expressing only CXCR4, CXCL12 binding to CXCR4 initiates signaling pathways typical of seven transmembrane receptors, including activation of heterotrimeric G proteins and recruitment of the cytosolic adapter protein β-arrestin 2. The CXCR4-β-arrestin 2 complex internalizes to endosomes, initiating β-arrestin-dependent signaling and ultimately leading to receptor degradation [9]. Conversely, CXCR7 is an atypical chemokine receptor that does not activate G proteins in response to CXCL12 [10]. CXCR7 functions in part as a chemokine decoy receptor for CXCL12, removing this chemokine from extracellular space and degrading it [11]–[13]. Functions of CXCR7 are enhanced by 10-fold higher binding affinity for CXCL12 relative to CXCR4 and constitutive internalization and recycling of CXCR7 to the cell membrane [12], [14]. In response to CXCL12, CXCR7 also signals through β-arrestin 2 dependent pathways on endosomes [3], [15].

Cells commonly co-express CXCR4 and CXCR7 under both normal and pathologic conditions, and studies strongly suggest that cells regulate levels of these receptors to respond to the environment and acquire new functions. For example, estrogen has been reported to increase expression of CXCR4 while reducing amounts of CXCR7 on breast cancer cells [16]. Activated macrophages increase mRNA and protein for CXCR7 while downregulating CXCR4, and platelets from patients with acute coronary artery disease increase CXCR7 while maintaining levels of CXCR4 [17], [18]. In addition, tumor-initiating cells from some brain cancer cell lines may preferentially express CXCR4, contrasting with more differentiated cancer cells with greater expression of CXCR7 [19]. Changes in numbers of CXCR7 versus CXCR4 receptors on cells may alter signaling pathways normally activated by CXCR4 alone, but reported effects are contradictory [20]–[22]. CXCR7 has been reported to either impair or enhance CXCL12-CXCR4 activation of G protein signaling. Co-expression of CXCR4 and CXCR7 also may increase β-arrestin-mediated signaling, although dynamics and distribution of β-arrestin 2 between CXCR4 and CXCR7 under basal and ligand-activated states remain unknown. Discordances among these studies with CXCR4 and CXCR7 may be due to factors including relative differences in ratios of CXCR4 and CXCR7 used by different authors.

Prior studies by our group and others have analyzed pairwise interactions of β-arrestin 2 with either CXCR4 or CXCR7 under basal conditions and in response to ligands such as CXCL12 [14], [21], [23]–[26]. These experiments lacked the capability to simultaneously quantify recruitment of β-arrestin 2 to each receptor in cells co-expressing both CXCR4 and CXCR7, precluding direct analyses of competition for this adapter protein. To overcome this limitation, we utilized a recently described dual color click beetle luciferase complementation assay for bioluminescence imaging of two different proteins interacting with a shared partner [27]. By fusing CXCR4 and CXCR7 to N-terminal fragments of click beetle red and green luciferases and β-arrestin 2 to the common C-terminal fragment, we could directly measure association of β-arrestin 2 with each receptor in different spectral windows. Dual color luciferase complementation also has the advantage of quantifying protein interactions in the same population of intact cells over time.

To capture complex dynamics of CXCL12-dependent recruitment of β-arrestin 2 in cells expressing CXCR4 (CXCR4^+^), CXCR7 (CXCR7^+^) or both (CXCR4^+^-CXCR7^+^), we used a systems biology approach combining dual color luciferase complementation imaging with computational modeling of receptor signaling and trafficking. We focused on recruitment of β-arrestin 2 to CXCR4 or CXCR7 since this is a common, early event in ligand-dependent activation of both receptors. Based on imaging data from cells expressing either CXCR4 or CXCR7, we developed and tuned a computational model that describes kinetics and magnitude of interactions with β-arrestin 2. This approach builds on advantages of computational models to advance understanding of complex kinetic events and non-linear pathways in signaling [28], [29]. Our systems biology approach successfully predicted perturbations in β-arrestin 2 recruitment to CXCR4 and CXCR7 in cells expressing both receptors and identified levels of β-arrestin 2 as a key control point in CXCL12-CXCR4 signaling. These findings elucidate how co-expression of CXCR4 and CXCR7 regulates β-arrestin 2 recruitment and underscore the power of an integrated computational modeling and quantitative imaging approach to investigate integrated functions of CXCL12, CXCR4, and CXCR7.

## Results

### Association of CXCR4 and CXCR7 with β-arrestin 2 in CXCR4^+^ and CXCR7^+^ cells

**Figure 1:**
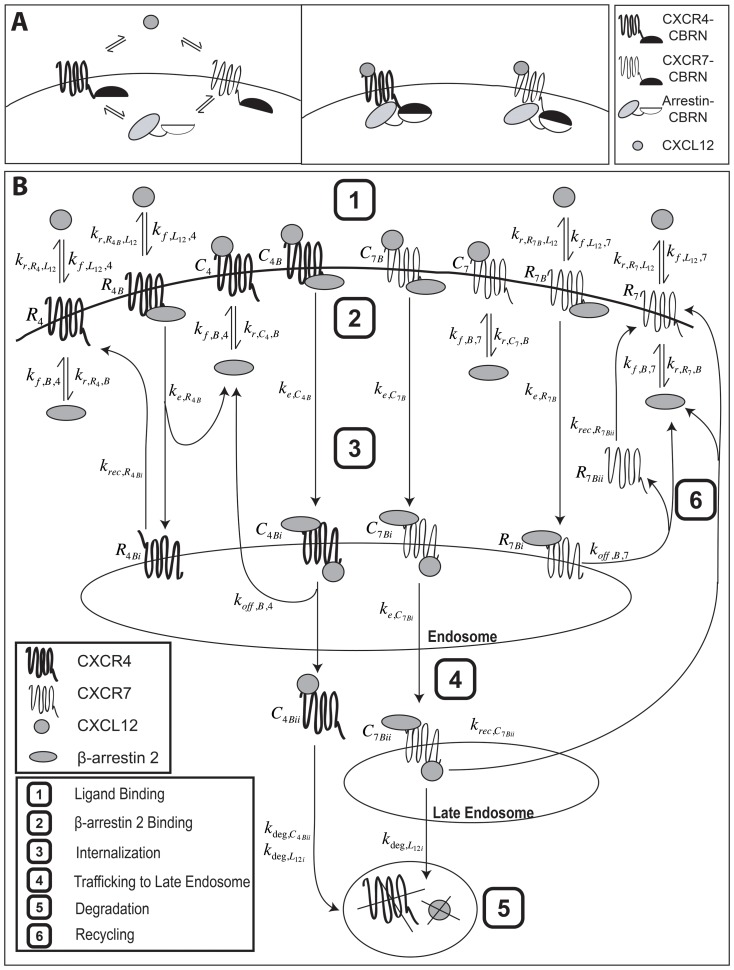
Diagrams of click beetle complementation reporters and computational model of β-arrestin 2 recruitment to CXCR4^+^ or CXCR7^+^. (A) Schematic of luciferase complementation reporters for CXCR4 or CXCR7 interaction with β-arrestin 2. (B) Model schematic of receptor dynamics for CXCR4^+^ cells (left) and CXCR7^+^ cells (right) with β-arrestin 2. Note that chematic does not distinguish between endogenous β-arrestin 2 and β-arrestin 2-CBC.

**Figure 2:**
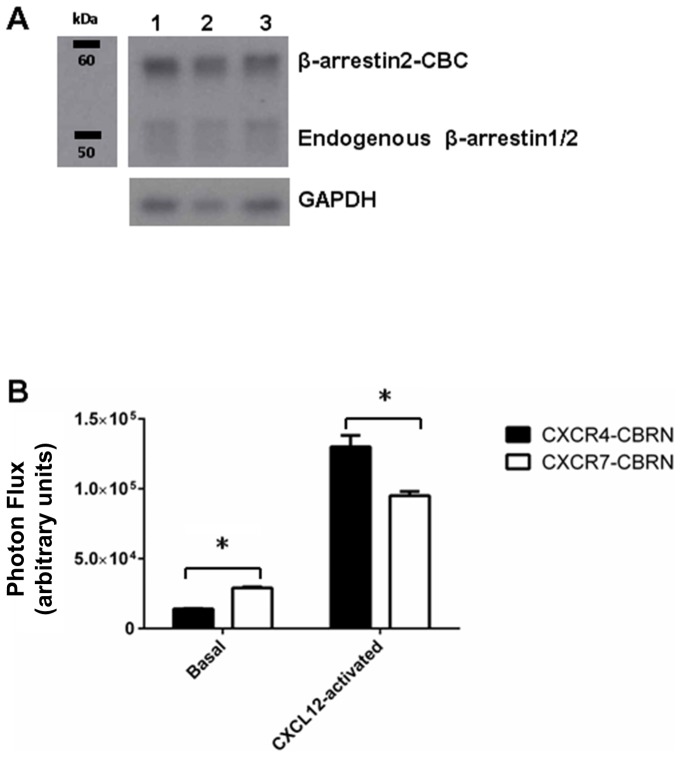
Luciferase complementation system reports on association of CXCR4 or CXCR7 with β-arrestin 2. (A) Expression of stably transduced β-arrestin 2-CBC and endogenous β-arrestin 1/2 in total lysates were detected by Western blot. Blots were stripped and re-probed for GAPDH as a loading control. Lane 1, CXCR4-CBRN/β-arrestin 2-CBC; lane 2, CXCR7-CBRN/β-arrestin 2-CBC; lane 3, CXCR4-CBRN/CXCR7-CBGN/β-arrestin 2-CBC. (B) Bioluminescence in CXCR4-CBRN/β-arrestin 2-CBC and CXCR7-CBRN/β-arrestin 2-CBC cells was measured under basal conditions and 18 minutes after adding 1000 ng/ml CXCL12-α. Graph shows mean values for photon flux arbitrary units + SEM for CXCR4+ or CXCR7+ cells (n = 4 per condition). *, significant difference.

To study interactions of CXCR4 and CXCR7 with β-arrestin 2, we used a combination of data derived from a luciferase complementation system based on green- and red-shifted variants of click beetle luciferase (Fig. 1A) and outputs from a computational model based on ordinary differential equations (Fig. 1B). In the luciferase complementation system, CXCR4 and CXCR7 are fused to the N-terminal fragment of click beetle red or green luciferase (CBRN or CBGN), while β-arrestin 2 is fused to the common C-terminal enzyme fragment (CBC). We initially transduced MDA-MB-231 breast cancer cells with β-arrestin 2-CBC, so all cells express the same levels of this fusion protein. We then transduced cells with either CXCR4 or CXCR7 fusions. We validated expression of β-arrestin 2-CBC and receptor fusions by Western blot and RT-PCR, respectively (Fig. 2A and Table A in File S1). Using spectral imaging of red bioluminescence, we showed significantly greater basal association of β-arrestin 2-CBC with CXCR7-CBRN than CXCR4-CBRN (p<0.01; Fig. 2B).

To quantify kinetics of ligand-dependent recruitment of β-arrestin 2 to each receptor, we treated reporter cells with increasing concentrations of CXCL12 from 0–1000 ng/ml and imaged cells every two min for 40 min and again at 90 min. We normalized data to values from cells treated with vehicle control at each time point. This approach focuses on relative increases in CXCL12-dependent recruitment of β-arrestin 2 to CXCR4 or CXCR7 and accounts for depletion of luciferin substrate over time.

**Figure 3:**
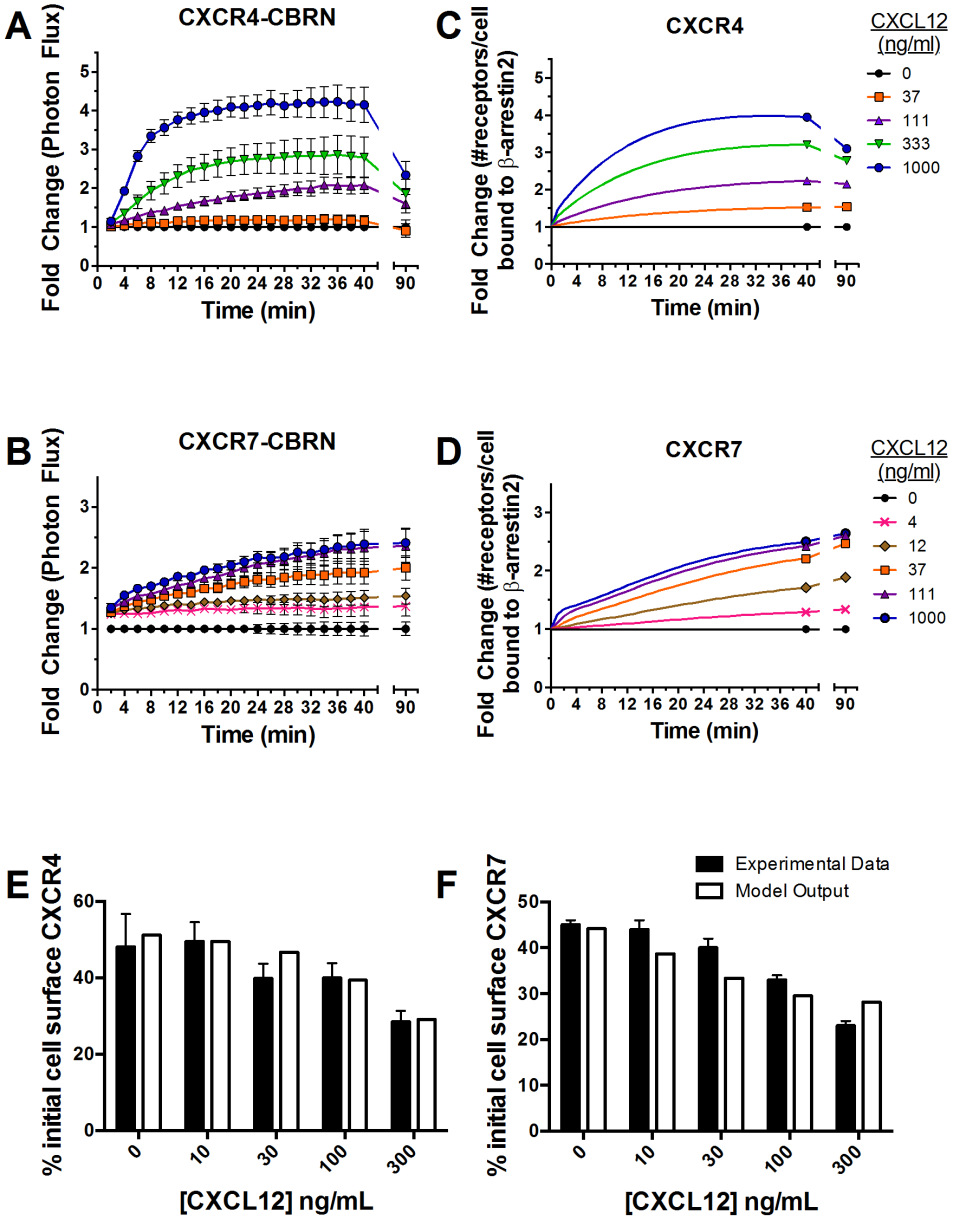
Kinetics of β-arrestin 2 recruitment to CXCR4 or CXCR7. (A and B) MDA-MB-231 breast cancer cells expressing CXCR4-CBRN/β-arrestin 2-CBC (A) or CXCR7-CBRN/β-arrestin 2-CBC (B) were treated with increasing concentrations of CXCL12-α (ng/mL) as denoted in the legend. Data were collected as photon flux units. Photon flux values for each time point then were normalized to values obtained for control cells not incubated with CXCL12 at each time point through 40 min and at 90 min. Data are expressed as mean values ± SEM for fold change relative to control (n = 4 per point). (C and D) Experimental data were used to tune parameters for a computational model describing numbers of receptors per cell bound to β-arrestin 2. Model outputs for CXCR4 (C) and CXCR7 (D) were plotted as fold change relative to cells not treated with CXCL12. (E, F) Internalization of cell surface CXCR4 (E) or CXCR7 (F) following 40 min or 30 min, respectively, of incubation with CXCL12 was measured by flow cytometry. Values for 0 ng/ml CXCL12 describe internalization of CXCR4 or CXCR7 in the absence of ligand. Experimental data for CXCR7 were replotted based on previously published results [12]. Model fits also are shown.

Association of CXCR4-CBRN with β-arrestin 2-CBC increased rapidly in a concentration-dependent manner after adding CXCL12 (Fig. 3A, Fig. A in File S1). As little as 37 ng/mL CXCL12 increased association of CXCR4 and β-arrestin 2 above basal levels, and 1000 ng/mL produced a 4-fold increase in bioluminescence. Interaction of CXCR4 and β-arrestin 2 peaked at ≈20–22 minutes for cells treated with 1000 ng/mL CXCL12, while plateau levels occurred slightly later for cells incubated with lower concentrations of CXCL12. Association of CXCR4-CBRN with β-arrestin 2-CBC decreased in a concentration-dependent manner by 90 min.

Cells expressing CXCR7-CBRN/β-arrestin 2-CBC also showed concentration-dependent increases in bioluminescence with only 4 ng/mL required to boost signal above basal levels (Fig. 3B, Fig. A in File S1). The difference in lower limits of detection for β-arrestin 2 recruitment by CXCR7 reflects greater affinity of this ligand-receptor pair relative to CXCL12-CXCR4 [14]. Ligand-dependent association of CXCR7 and β-arrestin 2 increased progressively over the full time course, reaching a maximum of 2.4-fold induction after 40 min with 1000 ng/mL CXCL12. Cells expressing CXCR7-CBRN exhibited more prolonged association with β-arrestin 2-CBC, staying constant through 90 min for all concentrations. After 90 min, ligand-dependent recruitment of β-arrestin 2 to CXCR7 was comparable to CXCR4.

### Describing kinetics of β-arrestin 2 recruitment and receptor internalization in CXCR4^+^ and CXCR7^+^ cells by computational modeling

We developed a data-driven computational model based on ordinary differential equations to describe receptor internalization and kinetics of CXCL12-dependent recruitment of β-arrestin 2 to either CXCR4 or CXCR7 in cells expressing a single complementation reporter (Fig. 1B, Tables 1-2; Table B in File S1). Consistent with experimental data, interaction of CXCR4 with β-arrestin 2 increases rapidly within 12–14 min, reaching a maximum of ≈4-fold above basal levels for 1000 ng/mL CXCL12 (Fig. 3C). The initial increase in recruitment to CXCR4 is due to increasing numbers of cell-surface ligand-bound CXCR4, which have a higher affinity for β-arrestin 2 than free CXCR4 (Fig. B in File S1). More prolonged β-arrestin 2 recruitment is due to an increase in the number of intracellular CXCR4 molecules bound to β-arrestin 2. The model also shows delayed kinetics of β-arrestin 2 recruitment to CXCR4 with lower concentrations of CXCL12. Association of CXCR4 and β-arrestin 2 decreased after 40 min for 1000 ng/mL CXCL12, declining to ∼75% of peak value by 90 min. At lower concentrations of CXCL12, recruitment increases at a slower rate and then plateaus.

**Table 1:** Description of species included in model and steady-state values in the absence of ligand.

Species	Description	Steady-state values in the absence of ligand for single-expressing cells*	Steady-state values in the absence of ligand for co-expressing cells**
*R­_4_* (#/cell)	Free cell-surface CXCR4	9.5×10^4^	1.3×10^5^
*R_7_* (#/cell)	Free cell-surface CXCR7	5.0×10^5^	5.1×10^5^
*L_12_* (nM)	Free extracellular CXCL12	0	0
*B_e_* (#/cell)	Free endogenous β-arrestin 2	5.0×10^5^–*R_4Be_* or 5.0×10^5^–*R_7Be_* - *R_7Bei_*	5.0×10^5^–*R_4Be_* or 5.0×10^5^–*R_7Be_* - *R_7Bei_*
*B_p_* (#/cell)	Free β-arrestin 2-CBC	1.5×[*B_e_*]	1.5×[*B_e_*]
*R_4Be_* (#/cell)	*R­_4_* bound to *B_e_*	5.8×10^3^	5.4×10^3^
*R_7Be_* (#/cell)	*R­_7_* bound to *B_e_*	6.5×10^4^	6.5×10^4^
*R_4Bp_* (#/cell)	*R­_4_* bound to *B_p_*	8.7×10^3^	8.0×10^3^
*R_7Bp_* (#/cell)	*R­_7_* bound to *B_p_*	9.7×10^4^	9.7×10^4^
*C_4_* (#/cell)	*R­_4_* bound to *L_12_*	0	0
*C_7_* (#/cell)	*R­_7_* bound to *L_12_*	0	0
*C_4Be_* (#/cell)	*R­_4Be_* bound to *L_12_*	0	0
*C_7Be_* (#/cell)	*R­_7Be_* bound to *L_12_*	0	0
*C_4Bp_* (#/cell)	*R­_4Bp_* bound to *L_12_*	0	0
*C_7Bp_* (#/cell)	*R­_7Bp_* bound to *L_12_*	0	0
*R_4Bei_* (#/cell)	Intracellular *R­_4Be_*	2.0×10^5^	1.8×10^5^
*R_7Bei_* (#/cell)	Intracellular *R­_7Be_*	1.0×10^5^	9.9×10^4^
*R_4Bpi_* (#/cell)	Intracellular *R­_4Bp_*	2.9×10^5^	2.7×10^5^
*R_7Bpi_* (#/cell)	Intracellular *R­_7Bp_*	1.5×10^5^	1.5×10^5^
*C_4Bei_* (#/cell)	Intracellular *C_4Be_*	0	0
*C_7Bei_* (#/cell)	Intracellular *C_7Be_*	0	0
*C_4Bpi_* (#/cell)	Intracellular *C_4Bp_*	0	0
*C_7Bpi_* (#/cell)	Intracellular *C_7Bp_*	0	0
*R_7Beii_* (#/cell)	*R_7Bei_* after *B_e_* dissociation	2.3×10^5^	2.3×10^5^
*R_7Bpii_* (#/cell)	*R_7Bpi_* after *B_p_* dissociation	3.5×10^5^	3.5×10^5^
*C_4Beii_* (#/cell)	*C_4Bei_* after *B_e_* dissociation	0	0
*C_4Bpii_* (#/cell)	*C_4Bpi_* after *B_p_* dissociation	0	0
*C_7Beii_* (#/cell)	*C_7Bei_* after trafficking to late endosomes	0	0
*C_7Bpii_* (#/cell)	*C_7Bpi_* after trafficking to late endosomes	0	0
*L_12i_* (#/cell)	Intracellular *L_12_*	0	0

*Values correspond to steady-state conditions in single-expressing cells where the total number of cell surface and intracellular receptors is 6×10^5^ and 1.5×10^6^ receptors/cell for CXCR4 and CXCR7, respectively. The total number of β-arrestin 2 molecules is 5.0×10^5^ and 7.5×10^5^ molecules/cell for endogenous β-arrestin 2 and β-arrestin 2-CBC, respectively. Receptor numbers are based on reasonable agreement with the data in Table C in File S1, the assumption that a large portion of the receptors are intracellular in the absence of ligand, and ability to fit internalization data (Fig. 3 E,F). β-arrestin 2 numbers are based on our data suggesting that the ratio of probe-labeled/endogenous β-arrestin 2 is ∼ 1.5 (Fig. 2A) and literature data (12).

**Values correspond to steady-state conditions in co-expressing cells where the total number of cell surface and intracellular receptors is 6×10^5^ and 1.5×10^6^ receptors/cell for CXCR4 and CXCR7, respectively. The total number of β-arrestin 2 molecules is 5.0×10^5^ and 7.5×10^5^ molecules/cell for endogenous β-arrestin 2 and β-arrestin 2-CBC, respectively.

**Table 2:** Description and values of parameters.

Parameter	Description	Value	Literature Values	Reference
*k_f,L12,4_* (nM^−1^s^−1^)	Forward rate constant of *L_12_* binding *R_4_ /R_4Be_ /R_4Bp_*	2.1×10^−3^ †	2.8–6.7×10^−3^	[44]
*k_f,L12,7_* (nM^−1^s^−1^)	Forward rate constant of *L_12_* binding *R_7_ /R_7Be_ /R_7Bp_*	1.4×10^−3^ ††	2.8–6.7×10^−3^	[44]-[46]
*k_f,B,4_* ((#/cell) ^−1^s^−1^)	Forward rate constant of *B_e_ /B_p_* binding *R_4_ /C_4_*	8.5×10^−9^ † (4.3×10^−5^ nM^−1^s^−1^)**	10^−8^–10^−6^	[47] * [48] ^∧^
*k_f,B,7_* ((#/cell) ^−1^s^−1^)	Forward rate constant of *B_e_* or *B_p_* binding *R_7_ /C_7_*	1.4×10^−8^ †† (7.1×10^−5^ nM^−1^s^−1^)**	10^−8^–10^−6^	[47] * [48] ^∧^
*K_D,R4,L12_* (nM)	Equilibrium dissociation constant of *L_12_* binding *R_4_*	40	2-27	[49], [50]
*K_D,R7,L12_* (nM)	Equilibrium dissociation constant of *L_12_* binding *R_7_*	0.84	0.2–0.4	[51]
*K_D,R4B,L12_* (nM)	Equilibrium dissociation constant of *L_12_* from *R_4Be_ /R_4Bp_*	Equation (1) in text		[38], [52]
*K_D,R7B,L12_* (nM)	Equilibrium dissociation constant of *L_12_* from *R_7Be_ /R_7Bp_*	Equation (2) in text		[38], [52]
*K_D,R4,B_* (#/cell)	Equilibrium dissociation constant of *B_e_ /B_p_* from *R_4_*	7.8×10^6^ † (1.5×10^3^ nM)**	10^4^–10^6^	[47] * [48] ^∧^
*K_D,R7,B_* (#/cell)	Equilibrium dissociation constant of *B_e_ /B_p_* from *R_7_*	2.3×10^6^ †† (4.5×10^2^ nM)**	10^4^–10^6^	[47] * [48] ^∧^
*K_D,C4,B_* (#/cell)	Equilibrium dissociation constant of *B_e_ /B_p_* from *C_4_*	5.1×10^6^ † (1.0×10^3^ nM)**	10^4^–10^6^	[47] * [48] ^∧^
*K_D,C7,B_* (#/cell)	Equilibrium dissociation constant of *B_e_ /B_p_* from *C_7_*	6.5×10^5^ †† (1.3×10^2^ nM)**	10^4^–10^6^	[47] * [48] ^∧^
*k_e,R4B_* (s^−1^)	*R_4Be_ /R_4Bp_* internalization rate constant	2.3×10^−3^	1–2×10^−3^	[50]
*k_e,R7B_* (s^−1^)	*R_7Be_ /R_7Bp_* internalization rate constant	3.9×10^−3^	1–2×10^−3^	[50]
*k_e,C4B_* (s^−1^)	*C_4Be_ /C_4Bp_* internalization rate constant	4.7×10^−3^ †	3×10^−3^	[53]
*k_e,C7B_* (s^−1^)	*C_7Be_ /C_7Bp_* internalization rate constant	2.1×10^−3^ ††	3×10^−3^	[53]
*k_off,B,4_* (s^−1^)	Dissociation rate constant of *B_e_/B_p_* from *C_4Bei_ /C_4Bpi_*	7.4×10^−4^ †		
*k_off,B,7_* (s^−1^)	Dissociation rate constant of *B_e_/B_p_* from *R_7Bei_ /R_7Bpi_*	2.5×10^−3^ ††		
*k_e,C7Bi_* (s^−1^)	Rate constant of trafficking of *C_7Bei_ /C_7Bpi_* to late endosomes	5.5×10^−4^ ††		
*k_rec,R4Bi_* (s^−1^)	*R_4Bei_ /R_4Bpi_* recycling rate constant	6.9×10^−5^ †	10^−4^–10^−3^	[54]
*k_rec,R7Bii_* (s^−1^)	*R_7Beii_ /R_7Bpii_* recycling rate constant	1.1×10^−3^ ††	10^−4^–10^−3^	[54]
*k_rec,C7Bii_* (s^−1^)	*C_7Beii_ /C_7Bpii_* recycling rate constant	2.8×10^−4^ ††	10^−4^–10^−3^	[54]
*k_deg,C4Bii_* (s^−1^)	*C_4Beii_ /C_4Bpii_* degradation rate constant	1.0×10^−4 ***^	10^−5^–10^−4^	[55]
*k_deg,L12i_* (s^−1^)	*L_12i_* degradation rate constant	1.0×10^−4 ***^	10^−4^–10^−3^	[55]
*n_4_* (#/well)	# CXCR4^+^ cells per well	4.0×10^4^ °		
*n_7_* (#/well)	# CXCR7^+^ cells per well	4.0×10^4^ °		
*n_47_* (#/well)	# CXCR4^+^-CXCR7^+^ cells per well	4.0×10^4^ °		
*V*(L)	Well volume	7.0×10^−5^ °		

? Fit to internalization and β-arrestin 2 binding data with CXCL12 and CXCR4 in CXCR4^+^ cells.

?? Fit to internalization and β-arrestin 2 binding data with CXCL12 and CXCR7 in CXCR7^+^ cells.

^*^Reference gives maximum rate of β-arrestin 2 binding as *k_f_[β]_total_* = 0.136 s^−1^. Assuming a range of 10^5^–5×10^6^ β-arrestin 2 per cell gives *k_f_*≈10^−8^–10^−6^ (#/cell) ^−1^s^−1^. Reference gives β-arrestin 2 dissociation rate constant as *k_r_*≈0.024 s^−1^, which gives a β-arrestin 2/receptor equilibrium dissociation constant *K_D_*≈10^4^–10^6^ (#/cell).

^**^Rate constants *(k)* and equilibrium dissociation constants *(K)* are converted from #/cell to their effective value in nM using: 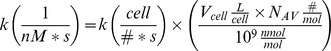 and

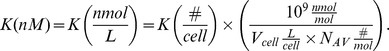

Cell volume (V_cell_) is assumed to be 8.4×10^−12^ L based on a spherical, 20 µm diameter cell.

^***^These parameters do not affect model output of fold change of β-arrestin bound (see Fig. A in File S1) but are included for completeness.

°Experimental conditions. Cells are assumed to grow to 2–3x above confluence at the time of plating.

∧Value was converted from units reported to these units by using Avogadro's number and cell volume from original paper.

In contrast, association of β-arrestin 2 with CXCR7 increases slowly throughout 40 min for all concentrations of CXCL12 (Fig. 3D). Interaction with β-arrestin 2 increases through 90 min in cells treated with 12 ng/mL or higher CXCL12, while recruitment maintains the same level by 90 min at lower concentrations. Similar to CXCR4, the initial increase in β-arrestin 2 recruitment to CXCR7 is due to an increase in the number of ligand-bound CXCR7 receptors on the cell surface, whereas later kinetics of β-arrestin 2 recruitment are governed by increasing intracellular pools of receptors bound to β-arrestin (Fig. B in File S1).

The model also describes experimental data for receptor internalization in the absence and presence of CXCL12 (Fig. 3E and 3F). These results establish that our computational model reproduces both CXCL12-dependent β-arrestin 2 recruitment to CXCR4 and CXCR7 and receptor internalization observed in dynamic biological systems.

### Predictions of β-arrestin 2 binding affinity, available β-arrestin 2, and available receptors

We can use the computational model to infer mechanisms that drive observed behavior. In the absence of CXCL12, we calculate the apparent equilibrium dissociation constant of β-arrestin 2 for CXCR4 (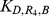) as over 3 times the value of the apparent equilibrium dissociation constant of β-arrestin 2 for CXCR7 (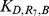) (1.5×10^3^ nM and 4.5×10^2^ nM, respectively; Table 2). Thus, CXCR7 has higher binding to β-arrestin 2 under basal conditions and must recruit more β-arrestin 2 following ligand addition to achieve the same fold-change value as CXCR4.

**Figure 4:**
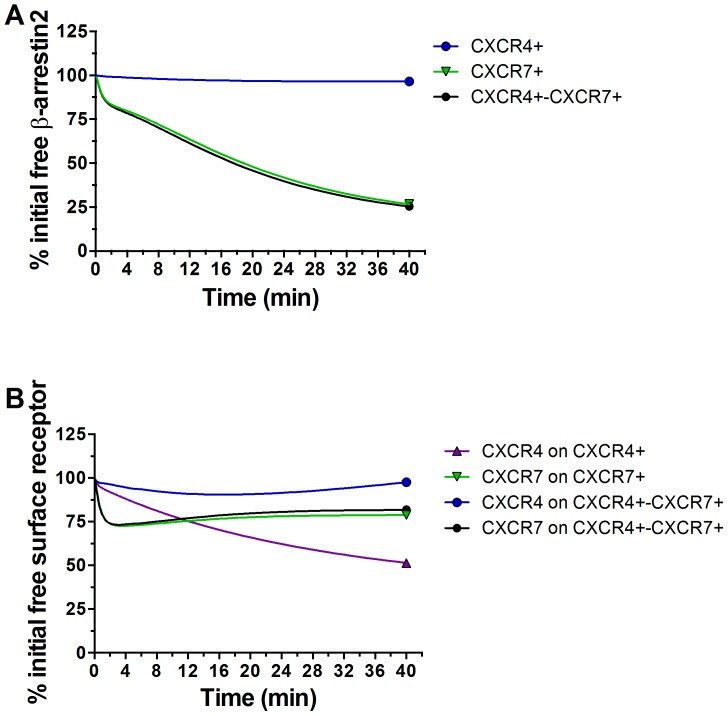
Modeling free β-arrestin 2 and free (unbound to β-arrestin 2) cell surface receptors over time. (A) Model output of the % of initial free β-arrestin 2 through 40 min in CXCR4^+^, CXCR7^+^, or CXCR4^+^-CXCR7^+^ cells treated with 1000 ng/mL CXCL12-α. (B) Model output of % of initial free (unbound to β-arrestin 2) cell surface receptors through 40 min in cells treated with 1000 ng/mL CXCL12. Legend denotes the specific receptor and cell type on which the receptor is expressed.

Recruitment of β-arrestin 2 to CXCR4^+^ cells in response to 1000 ng/mL CXCL12 peaks at ≈ 20-22 min and plateaus or decreases through 90 min at all ligand concentrations. We predict an excess of β-arrestin 2 throughout this time (Fig. 4A). In contrast, the number of CXCR4 receptors able to bind β-arrestin 2 decreases to ≈50% of the initial value 40 min after adding CXCL12 (Fig. 4B). Internalized, ligand-bound CXCR4 is degraded, decreasing numbers of cell surface receptors and subsequently reducing the rate of β-arrestin 2 binding. Therefore, β-arrestin 2 recruitment in CXCR4^+^ cells is limited by the number of cell-surface receptors unbound to β-arrestin 2 and not the amount of β-arrestin 2.

In CXCR7^+^ cells, CXCL12 increases association of β-arrestin 2 with CXCR7 throughout a 40 min experiment (Fig. 4A)**.** Our model predicts the number of CXCR7 receptors unbound to β-arrestin 2 initially decreases after adding 1000 ng/mL CXCL12 and then partially recovers as internalized receptors recycle to the cell surface (Fig. 4B). Recycled CXCR7 rebinds β-arrestin 2, contributing to a progressive increase in interactions over time. Internalized CXCR7 also remains associated with β-arrestin 2, so complexes of CXCR7 and β-arrestin 2 accumulate intracellularly and continue to produce bioluminescence.

### CXCR7 decreases recruitment of β-arrestin 2 to CXCR4 in cells co-expressing both receptors (CXCR4^+^-CXCR7^+^)

**Figure 5:**
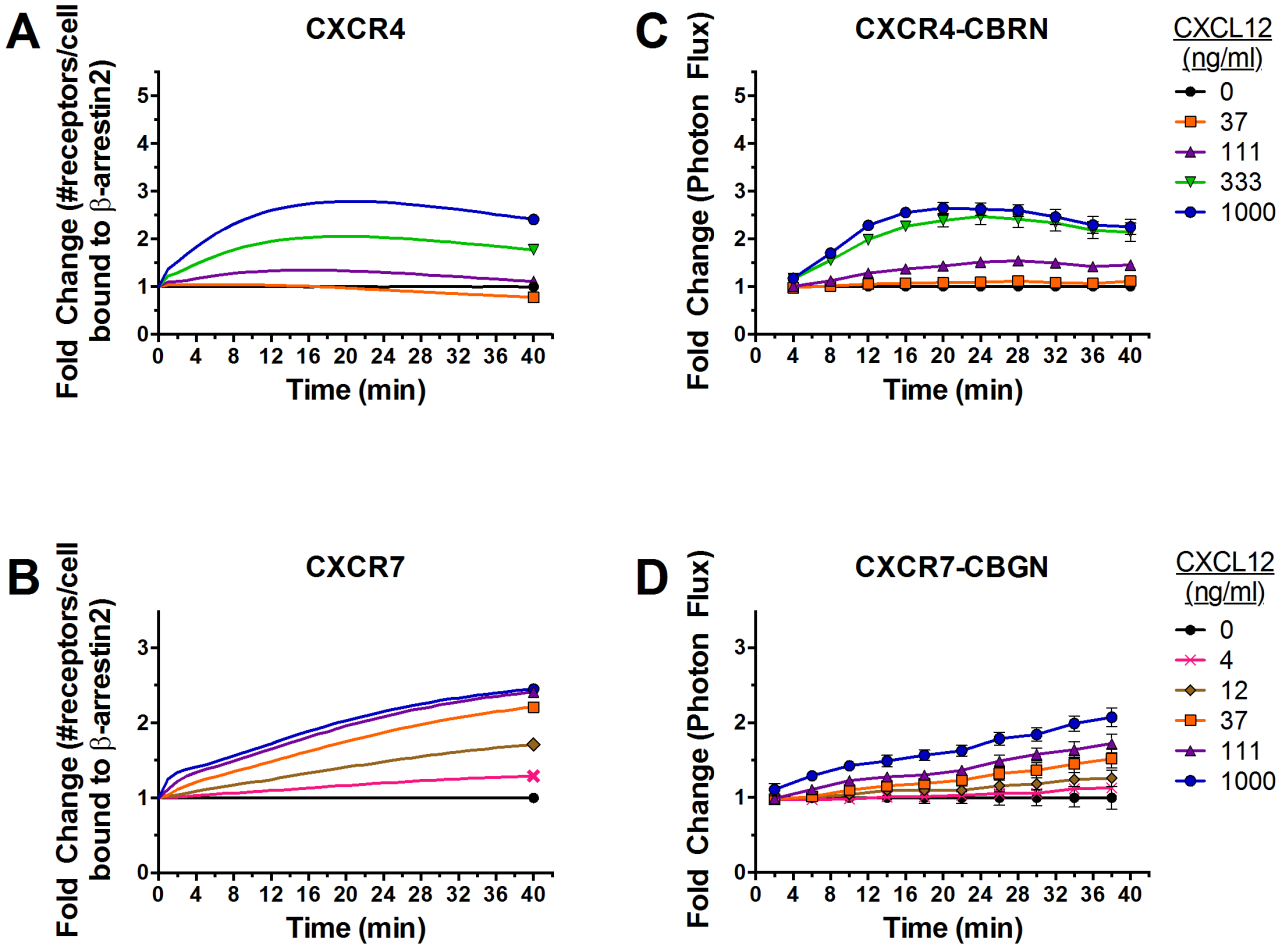
CXCR7 limits interaction of CXCR4 and β-arrestin 2 in CXCR4^+^-CXCR7^+^ cells. (A and B) Model outputs for CXCL12-dependent recruitment of β-arrestin 2 specifically to CXCR4 (A) or CXCR7 (B) in CXCR4^+^-CXCR7^+^ MDA-MB-231 cells. (C and D) Experimental data for recruitment of β-arrestin 2-CBC to CXCR4-CBRN (C) or CXCR7-CBGN (D) in CXCR4^+^-CXCR7^+^ cells. Legend shows concentrations of CXCL12-α used for models and experimental data. Data were graphed as mean values ± SEM for fold change in bioluminescence relative to untreated cells as in Figure 1 (n = 4 per experimental point).

We next used the computational model to predict how co-expression of CXCR4 and CXCR7 on the same cell affects recruitment of β-arrestin 2 to each receptor. Simulated experiments using receptor numbers typical of our cells show that maximum fold change for recruitment of β-arrestin 2 to CXCR4 decreases at all concentrations of CXCL12 in CXCR4^+^-CXCR7^+^ cells (Fig. 5A). Compared with our model output for CXCR4^+^ cells, peak fold-induction for β-arrestin 2 recruitment to CXCR4 decreases by ∼30% in CXCR4^+^-CXCR7^+^ cells with a more pronounced decrease over time. Conversely, the model predicts only a slight reduction in maximum fold-change in β-arrestin 2 association with CXCR7 and minimal effect on progressive increase in signal over time (Fig. 5B).

Our model points to a likely explanation for decreased recruitment of β-arrestin 2 to CXCR4 in cells co-expressing CXCR7. In contrast to single-receptor simulations, cell surface CXCR4 remains elevated through 40 min in co-expression simulations and does not limit β-arrestin 2 recruitment (Fig. 4B). Instead, the limiting factor is the amount of β-arrestin 2 available for binding. Free β-arrestin 2 decreases substantially in CXCR4^+^-CXCR7^+^ cells, paralleling the decrease in free β-arrestin 2 in CXCR7^+^ cells (Fig. 4A). The decrease in free β-arrestin 2 is due to two factors: 1) ∼ 50-fold greater affinity of CXCL12 for CXCR7 than for CXCR4; and 2) ∼ 8-fold greater affinity of β-arrestin 2 for ligand-bound CXCR7 than for ligand-bound CXCR4 (see Table 2). These factors limit the amount of β-arrestin 2 available to bind CXCR4, as CXCR7 literally “steals” β-arrestin 2 away from the other receptor. Internalized CXCR7 remains bound to β-arrestin 2, further limiting amounts of free β-arrestin 2. Collectively, these data demonstrate that CXCR7 limits availability of free β-arrestin 2, thereby diminishing CXCL12-dependent association of CXCR4 with this scaffolding protein.

To test model predictions, we quantified interaction of β-arrestin 2-CBC with CXCR7-CBGN or CXCR4-CBRN in cells expressing both receptors. Maximum signal for recruitment of β-arrestin 2 to CXCR4 increased by only ≈2.7-fold above control, representing an ≈35% decrease relative to cells with only CXCR4 (Fig. 5C). Detectable recruitment of β-arrestin 2-CBC to CXCR4-CBRN required 111 ng/mL CXCL12, which was substantially greater than the concentration of 37 ng/mL CXCL12 needed to increase signal above baseline in CXCR4^+^ cells. Additionally, both experimental and modeling outputs showed delayed, less sustained recruitment of β-arrestin 2-CBC to CXCR4-CBRN in CXCR4^+^-CXCR7^+^ cells (compare initial slopes for reporters in Fig. 3A and 5C).

By comparison, CXCR4 had minimal effects on interaction of CXCR7 with β-arrestin 2. Relative to CXCR7^+^cells, bioluminescence from CXCR7-CBGN and β-arrestin 2-CBC decreased minimally by 15% in CXCR4^+^-CXCR7^+^ cells treated with 1000 ng/mL CXCL12 (Fig. 5D). Co-expression of CXCR4 modestly increased the amount of CXCL12 needed to generate signal for CXCR7-CBGN and β-arrestin 2-CBC from 4 ng/mL to 12 ng/mL. Recruitment of β-arrestin 2 to CXCR7 in dual reporter cells increased over 40 min, which did not differ from kinetics measured in cells expressing only CXCR7.

### Levels of β-arrestin 2 control CXCL12-dependent association with CXCR4 on CXCR4^+^-CXCR7^+^ cells

**Figure 6:**
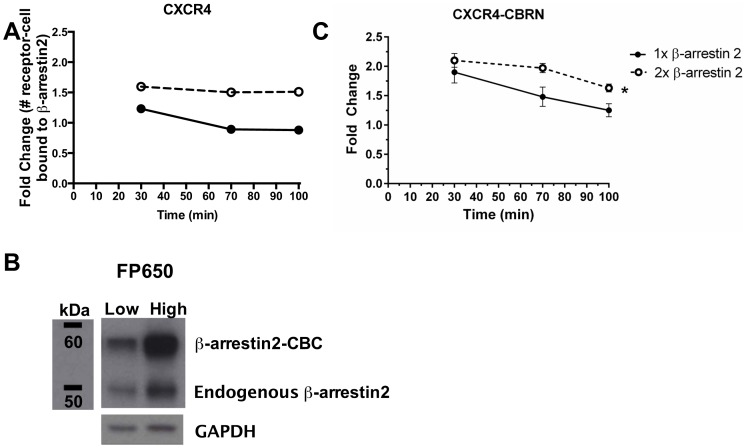
Overall levels of β-arrestin 2 limit interaction of CXCR4 and β-arrestin 2 in CXCR4^+^-CXCR7^+^ cells. (A to C) Figures display that over-expressing β-arrestin 2 increases ligand-induced β-arrestin 2-CBC recruitment to CXCR4-CBRN in CXCR4^+^-CXCR7^+^ cells at extended times. (A) Model output is plotted as fold change in the number of receptors bound to β-arrestin 2 through 100 min after treatment with 111 ng/mL CXCL12 normalized to untreated cells at each time-point. (B) Western blot for β-arrestin 2 in cells sorted for high and low levels of fluorescence from FP650. GAPDH is shown as a loading control. (C) Experimental data for β-arrestin 2-CBC recruitment to CXCR4-CBRN in CXCR4^+^-CXCR7^+^ cells graphed as mean values ± SEM for fold change of bioluminescence relative to vehicle control at 30, 70, and 100 min after treatment with 111 ng/mL CXCL12. *, significant difference determined by two-way ANOVA.

Model predictions and experimental data show that fold-change in β-arrestin 2 recruitment to CXCR4 in cells co-expressing both receptors decreases compared with cells expressing only CXCR4. The model identified the amount of β-arrestin 2 as the limiting factor, suggesting that increasing β-arrestin 2 in CXCR4^+^-CXCR7^+^ cells should alleviate suppression of β-arrestin 2 recruitment to CXCR4. Using our model, we predicted that increasing β-arrestin 2-CBC by 2-fold (designated as “2x” β-arrestin 2) at 111 ng/mL CXCL12 would significantly prolong β-arrestin 2 recruitment and elevate the fold-change value (Fig. 6A). Cells with 2x β-arrestin 2 maintained higher levels of association with CXCR4 than 1x β-arrestin 2 cells through 100-min. By this time, cells with 1x β-arrestin 2 returned to basal levels, whereas cells with 2x β-arrestin 2 maintained association with CXCR4 above baseline.

To validate model predictions, we sorted cells for high or low expression of β-arrestin 2-CBC based on fluorescence from FP650. Western blotting showed that cells with high FP650 fluorescence expressed ∼2-fold more β-arrestin 2-CBC than cells with low FP650 (Fig. 6B). Endogenous β-arrestin 2 levels were also higher in cells sorted for high FP650 intensity, which may be due to incomplete transcription of β-arrestin 2-CBC or post-translational cleavage of the CBC domain. However, recruitment kinetics of cells with low FP650 and unsorted cells showed indistinguishable recruitment kinetics, so parameters were conserved (Fig. C in File S1). Experiments with cells expressing high or low levels of β-arrestin 2-CBC validated overall patterns of model predictions (Fig. 6C; see Fig. D in File S1 for raw photon flux data). For cells with 2x β-arrestin 2, treatment with 111 ng/mL CXCL12 produced more sustained association of CXCR4 and β-arrestin 2 through 100 min with significantly greater signal at 70 and 100 min (p<0.05). Only 2x β-arrestin 2 cells also maintained complementation signal significantly above baseline through 100 min. These results verify that β-arrestin 2 levels significantly affect both kinetics and magnitude of recruitment to CXCR4 in cells co-expressing CXCR7. Receptor numbers are also predicted to affect the kinetics and magnitude of recruitment to CXCR4 and CXCR7 (Fig. E in File S1), suggesting that quantitative modulation of β-arrestin 2 recruitment is possible. These conclusions hold true in an expanded sensitivity analysis (Fig. F in File S1) that varies CXCR4, CXCR7, and β-arrestin 2 levels from more physiological values (10^3^ molecules/cell) to the lower limit of the overexpression system (10^6^ molecules/cell).

## Discussion

Precise spatial and temporal control of CXCL12 signaling is essential for normal development and physiology. CXCL12 signaling regulates chemotaxis and homing of stem cells to sites of injury, while perturbations of CXCL12 signaling through CXCR4 and/or CXCR7 drive pathogenesis of diseases such as cancer. Initial studies of CXCL12 signaling focused solely on chemokine receptor CXCR4. However, discovery of CXCR7 as a second receptor for CXCL12 means that biologic effects of this chemokine represent integrated output(s) of both CXCR4 and CXCR7. Prior studies indicate that functions of the CXCL12/CXCR4/CXCR7 axis are sensitive to expression of CXCR4 and CXCR7 on 1) separate populations of cells in the same tissue or organ; and 2) the same cell type [20], [30], [31]. Particularly for cells that co-express both CXCR4 and CXCR7, only limited information exists about how each receptor affects activation by CXCL12. To understand integrated functions of CXCR4 and CXCR7 and control these pathways for therapy, there is an unmet need to establish molecular mechanisms of CXCL12-dependent activation of one or both receptors on the same cell.

We developed a systems biology approach to investigate dynamics of β-arrestin 2 recruitment to CXCR4 and/or CXCR7. This approach combines real-time, multi-spectral luciferase complementation imaging with a data-driven computational model. Using cells expressing complementation reporters for β-arrestin 2 and either CXCR4 or CXCR7, we demonstrated that CXCL12 caused rapid, concentration-dependent recruitment of β-arrestin 2 to CXCR4 that peaked within 10–20 min and slowly diminished through 90 min. By comparison, ligand-dependent interaction of CXCR7 with β-arrestin 2 increased through 90 min with fold-induction over basal levels comparatively less than CXCR4. These data are consistent with prior studies done by our group and others categorizing CXCR4 and CXCR7 as class A and B seven transmembrane receptors based on transient and sustained association with β-arrestin 2, respectively [12], [14], [23], [32], [33]. We devised and tuned model parameters using data from cells expressing reporters for either CXCR4 or CXCR7 signaling. The resultant model closely reproduced the differing magnitude and kinetics of CXCR4 or CXCR7 association with β-arrestin 2 in cells expressing only one receptor, establishing that the model captures dynamics of this early step in receptor activation.

Through computational modeling and experiments, we established that CXCR7 wins the “competition” for CXCL12-dependent recruitment of β-arrestin 2 in cells that co-express both CXCR4 and CXCR7. Expression of CXCR7 on the same cells decreases the magnitude and duration of β-arrestin 2 recruitment to CXCR4 and elevates the concentration of CXCL12 required to produce a signal above basal levels. By comparison, co-expression of CXCR4 only minimally affected ligand-dependent recruitment of β-arrestin 2 to CXCR7. These outcomes occur because CXCR7 effectively sequesters β-arrestin 2 from CXCR4 in cells with both receptors. As predicted by computational modeling, increasing β-arrestin 2 partially overcomes suppressive effects of CXCR7 on recruitment of β-arrestin 2 to CXCR4. These results underscore interdependent effects of CXCR4 and CXCR7 on responses to CXCL12 and establish β-arrestin 2 as a key control point in these signaling pathways [21]. Receptor numbers also are predicted to affect the magnitude of recruitment to CXCR4 and CXCR7 (Fig. E and Fig. F in File S1), suggesting that quantitative modulation of both the absolute amount and fold-change of β-arrestin 2 recruitment is possible.

Our results for β-arrestin 2 recruitment and downstream signaling provide a quantitative, molecular mechanism to explain prior studies showing that CXCR7 may shift signaling toward β-arrestin 2 in cells that also express CXCR4. Décaillot et al reported that co-expression of CXCR4 and CXCR7 increased co-immunoprecipitation of β-arrestin 2 with CXCR7, potentiating β-arrestin 2-dependent signaling to MAPK pathways such as ERK1/2 and p38 while limiting signaling mediated by G proteins [21]. Sierro et al also demonstrated that co-expression of CXCR4 and CXCR7 eliminated early activation of ERK1/2 and produced sustained activation of these kinases in response to CXCL12, a characteristic feature of signaling mediated by β-arrestin 2 [20]. Co-expression of CXCR7 with CXCR4 also augmented intracellular calcium flux in response to CXCL12. Cell surface CXCR4 remains elevated in cells that co-express CXCR7, which could potentiate CXCL12-CXCR4 signaling to G proteins. Further modeling and experimental data are needed to establish effects of CXCR7 on the magnitude and duration of CXCR4 coupling to different downstream effectors in distinct contexts.

While our experimental and computational models include multiple parameters that control CXCL12 signaling, we recognize there are additional levels of complexity in this signaling pathway. We are limited by our luciferase complementation technology to quantifying two pairs of protein interactions based on green and red spectral variants of click beetle luciferase. To integrate other determinants of CXCL12 signaling such as chemokine or receptor dimers, we currently are working to incorporate additional complementation systems based on *Gaussia* or *Renilla* luciferases. These new data then will drive incorporation of additional elements into the computational model as needed to accurately describe and predict additional components of CXCL12/CXCR4/CXCR7 signaling.

## Conclusions

We have developed an integrated experimental and computational approach to quantify, describe, predict, and validate dynamics of CXCL12 signaling through CXCR4 and CXCR7 in living cells in real time. Through this approach, we have defined interdependent effects of CXCR4 and CXCR7 on recruitment of β-arrestin 2, a key node in this signal transduction pathway. In cells co-expressing both receptors, CXCL12 drives recruitment of β-arrestin 2 to CXCR7 and limits association of this scaffolding protein with CXCR4. Furthermore, we predicted and verified that amounts of β-arrestin 2 critically determine differences in CXCL12-association with CXCR4 versus CXCR7. Since the click beetle luciferase complementation reporter is compatible with high throughput assays, these reporter cells also could be used to screen libraries for molecules that target rate-limiting steps in CXCL12 signaling identified by modeling. The same reporter cells then can be used for imaging studies in living mice, allowing us to refine our computational model based on *in vivo* data. The molecular imaging and mathematical systems developed in this work will ultimately reveal how CXCL12 signaling pathways function in normal physiology and disease and facilitate ongoing efforts to control these pathways therapeutically.

## Methods

### Plasmids and lentiviruses

We used N-terminal and C-terminal fragments of click beetle green and red luciferases (Promega) comprising amino acids 2–413 and 395–542, respectively, for each spectral variant [27]. We designated N-terminal fragments as CBGN and CBRN for click beetle red and green, respectively, which confer spectral characteristics of each luciferase. The common C-terminal fragment (CBC) complements with either N-terminal fragment.

To sort transduced cell populations, we modified lentiviral vector FUGW to replace green fluorescent protein with mTagBFP, nuclear-localized citrine, or FP650 [34]. We cloned β-arrestin 2-CBC into the vector with FP650. We inserted CBGN fusions for CXCR4 or CXCR7 into a vector with co-expressed mTagBFP, and CBRN fusions were cloned into a vector with nuclear citrine. PCR primers used for cloning procedures are shown in Supplemental Methods in File S1. Amplified products were confirmed by DNA sequencing.

### Cells

We cultured MDA-MB-231 cells (ATCC) in DMEM (Life Technologies) with 10% serum, 1% glutamine, and 0.1% penicillin/streptomycin. We transduced 231 cells with lentiviruses at low multiplicity of infection for various click beetle complementation constructs as described previously [35]. We performed the first round of transduction with β-arrestin 2-CBC and sorted cells based on co-expressed FP650. Subsequent transductions added CXCR4 and CXCR7 fusions with CBGN, CBRN, or both. For cells co-expressing both receptors, we paired CXCR4-CBRN with CXCR7-CBGN or the reverse spectral combination. Since both spectral combinations performed comparably, we show data only for the CXCR4-CBRN and CXCR7-CBGN pair. We sorted transduced cell populations for mTagBFP or nuclear citrine in CBGN or CBRN constructs, respectively.

### Click beetle luciferase complementation for CXCR4 or CXCR7 interaction with β-arrestin 2

MDA-MB-231 human breast cancer cells stably expressing CXCR4-CBRN, CXCR7-CBRN, CXCR4-CBRN and CXCR7-CBGN, or 231 control cells were seeded at 1.5×10^4^ cells per well in 96 well black-wall plates. All cell lines except for 231 control cells also express β-arrestin 2-CBC. Cells were grown at 37°C for 2 days before assays. We gently aspirated medium from wells and replaced it with 50 µL phenol red free DMEM (Life Technologies) with 0.2% media grade probumin (Celliance) 30 min before imaging. We added 7 µL of a 15 mg/mL luciferin stock and then incubated cells for 5 min before adding CXCL12. Immediately before imaging, we added 14 µL phenol red free DMEM containing 0.2% probumin and increasing concentrations of synthetic CXCL12-α (R&D Systems). We acquired a series of 20 images with large binning, 2 minute exposure, and open filter on an IVIS 100 (Perkin Elmer) for plates containing 231-CXCR4-CBRN, 231-CXCR7-CBRN or 231-control cells. For cells expressing both green and red click beetle complementation reporters 231-(CXCR4-CBRN)-(CXCR7-CBGN), we obtained 20 images with large binning and 2 minute exposure, alternating between 530–550 nm or 690–710 nm emission filters (IVIS 200, Perkin Elmer). For longer time course data points, cells were maintained at 37°C and 5% CO_2_, and imaged again at 90 min. To determine relative induction of bioluminescence, we normalized bioluminescence for wells treated with CXCL12 to cells incubated with vehicle control at each time point (n = 4 per condition). Data were graphed as mean values ± standard error of the mean (SEM).

### Flow cytometry

We analyzed cell surface CXCR4 or CXCR7 by flow cytometry using monoclonal antibodies 12G5 (R&D Systems) and 11G8 (gift of ChemoCentryx), respectively [36]. We measured receptor expression by mean fluorescence intensity. We performed flow cytometry experiments for internalization of cell surface CXCR4 using monoclonal antibody 12G5 as described previously for internalization of CXCR7 [12]. Control cells were incubated without CXCL12 to quantify ligand-independent receptor internalization.

To obtain cell populations with high and low levels of β-arrestin 2-CBC, we sorted cells by fluorescence from co-expressed FP650. We collected cells with the top and bottom 10% of fluorescence intensities. We verified that these cell populations remained stable by repeating flow cytometry four days later.

### Western blotting

We analyzed endogenous β-arrestin 1 and 2 and transduced β-arrestin 2 in total cell lysates by Western blotting with a rabbit mAb (Cell Signaling) and an anti-rabbit secondary antibody conjugated with horse radish peroxidase (Cell Signaling). Primary and secondary antibody dilutions were 1∶1,000 and 1∶10,000. We detected bound antibody complexes with an ECL Plus kit (Amersham).

### Model

We developed a computational model to investigate dynamics of β-arrestin 2 recruitment to CXCR4 and CXCR7 (Fig. 1B). Events and pathways included are CXCL12 binding, β-arrestin 2 recruitment, internalization, recycling, and degradation. We include only β-arrestin 2 because experiments show significantly more association between β-arrestin 2 and CXCR4 and CXCR7 than between β-arrestin 1 and either receptor in the presence of CXCL12 [23]. The model includes two pools of β-arrestin 2, endogenous and reporter fusion to CBC, and receptors must bind β-arrestin 2 to internalize. We assume all internalized CXCL12 is degraded and all β-arrestin 2 is recycled following dissociation from internalized receptors [12]. Synthesis of receptors and β-arrestin 2 is assumed negligible during the timescale of the experiments [12].

CXCR4 is a type A receptor, transiently binding β-arrestin 2 [14]. In the absence of CXCL12, CXCR4 constitutively binds β-arrestin 2 [23]. We assume that β-arrestin 2 dissociates from CXCR4 not bound to ligand during internalization and that these receptors recycle to the cell surface. Receptors bound to ligand remain associated with β-arrestin 2 during internalization; following internalization, β-arrestin 2 dissociates and receptors are routed for degradation [12], [37].

CXCR7 is a type B receptor, tightly binding β-arrestin 2 [14]. Therefore, we assume that β-arrestin 2 remains associated with CXCR7 during internalization. All internalized CXCR7 is ultimately recycled, but receptors unbound and bound to ligand follow distinct routes following internalization. CXCR7 not bound to CXCL12 is directly recycled. However CXCL12-bound CXCR7 first trafficks to late endosomes before recycling as CXCL12 has been shown to slow receptor recycling [12]. We assume that β-arrestin 2 remains bound to CXCR7 through trafficking to late endosomes as recycling of CXCR7 has kinetics similar to dissociation of β-arrestin 2 [12].

The mathematical model consists of coupled nonlinear ordinary differential equations based on mass action kinetics (Tables 1-2, Table B in File S1). Equations were solved using ode45 in MATLAB (The MathWorks, Natick, MA).

### Model Parameter Values and Initial Conditions

We obtained initial estimates of parameter values from literature and our own data (Table 2). We set identical receptor binding and dissociation rate constants for endogenous β-arrestin 2 and β-arrestin 2-CBC.

Assuming no input of energy into the system, a thermodynamic relationship exists among the apparent equilibrium dissociation constants of free receptor for ligand, free receptor for β-arrestin 2, ligand-bound receptor for β-arrestin 2, and β-arrestin 2-bound receptor for ligand [38], [39]:
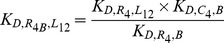
(1)

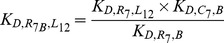
(2)


Model simulations begin with steady-state values of all species in the absence of ligand (Table 1). We estimated total amounts of cell-surface CXCR4 and CXCR7 on CXCR4^+^, CXCR7^+^, and CXCR4^+^-CXCR7^+^ cells in the absence of ligand by quantitative receptor binding assays (Supplementary Methods and Table C in File S1). The number of cell surface receptors for CXCR4 and CXCR7 are comparable to those reported previously for cells that express these receptors endogenously or through gene transfer [40], [41]. The total amount of endogenous β-arrestin 2 was determined to be on the same order of magnitude as the number of receptors because internalization of CXCR7 proceeds through β-arrestin 2 binding, and experiments have shown >50% internalization of CXCR7 [12]. The amount of β-arrestin 2-CBC relative to endogenous β-arrestin was measured by Western blotting (Fig. 2A).

### Fitting Model to Data

The computational model was simultaneously fit to experimental data on both β-arrestin 2-CBC recruitment (Fig. 3A,B) and receptor internalization (Fig. 3E,F) resulting from: (1) CXCL12 binding to CXCR4^+^ cells or (2) CXCL12 binding to CXCR7^+^ cells. To compare model output to these measurements, we calculated the fold change in β-arrestin 2-CBC recruitment for CXCR4^+^ cells at each time-point (t) and for each ligand concentration (L) as: 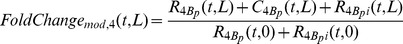
(3)and for CXCR7^+^ cells as:
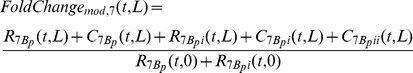
(4)


To compare model output to experimental data on receptor internalization, we first initialized the model with a total number of receptors and β-arrestin 2, and ran the simulations in the absence of ligand to steady-state. This step calculated the number of cell surface and internalized receptors at t = 0. All cell surface receptors were then mathematically differentiated from internalized receptors and internalization of these cell-surface receptors was tracked over time. For CXCR4, this was calculated as: 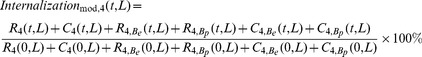
(5)and for CXCR7 this was calculated as:
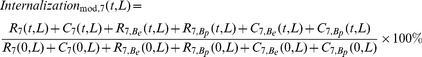
(6)where the species in these equations represent only receptors that are initially on the cell-surface.

See Table 1 for symbol descriptions.

Goodness of fit was assessed by calculating the sum of squared differences between model output and experimental data for β-arrestin 2 recruitment over time:
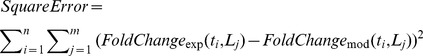
(7)and between model output and experimental data for receptor internalization over time:
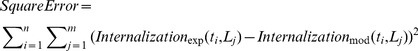
(8)where n is the number of ligand concentrations tested and m is the number of time-points analyzed for each ligand concentration. For CXCR4, the square error in β-arrestin 2 recruitment was compared at 6 different time-points (sufficient to reproduce the shape of the data) between 0 and 90 minutes and for 5 different concentrations of CXCL12 and the square error in internalization was compared at 1 time-point for 5 different concentrations of CXCL12. This gave a total of 35 data points for model fitting to CXCR4 data. For CXCR7, the square error in β-arrestin 2 recruitment was compared at 6 different time-points (sufficient to reproduce the shape of the data) between 0 and 90 minutes and for 6 different concentrations of CXCL12 and the square error in internalization was compared at 1 time-point for 5 different concentrations of CXCL12. This gave us a total of 41 points for model fitting to CXCR7 data.

To find the best fit, several rounds of Latin Hypercube Sampling (LHS) were used to sample the parameter space [42] with model simulations carried out for each of 1000 parameter sets for each of CXCR4 and CXCR7 in each round. The total square error, calculated as the sum of equations (7) and (8) above, was calculated for each simulation. We initially varied 11 parameters for CXCR4 and 12 parameters for CXCR7, varying parameters +/− an order of magnitude from the literature estimates listed in Table 2. The parameter set that resulted in the smallest total square error each for CXCR4 and CXCR7 was chosen (Table 2) and is used to generate the computational model portions of Figures 3–6. We also determined which parameters most affected model output using uncertainty and sensitivity analysis via calculation of Partial Rank Correlation Coefficients (PRCC) [28], [43] (Table D in File S1). For N = 1000 runs, parameters with a PRCC >0.09 or <−0.09 and a p-value <0.01 were considered significantly different from zero. As expected, some parameters (e.g. binding parameters) play a major role at early time points, while others (e.g. internalization and recycling parameters) are more significant at later time points, reinforcing the need to include biological processes operating over multiple time frames in the model.

### Statistics

We defined statistical significance as p <0.05 based on unpaired t-test comparisons with Welch's correction or two-way ANOVA on GraphPad Prism 5 software. Presented figures are representative of at least three independent experiments for all conditions.

## Supporting Information

File S1 **Contains the following files: Figure A:** Photon flux values for (A) CXCR4-CBRN and (B) CXCR7-CBGN corresponding to Fig 3A, 3B. **Figure B:** Molecular Species Contributing to Beta-arrestin 2 Recruitment. **Figure C:** Recruitment kinetics of 2x β-arrestin 2, 1x β-arrestin 2 and parental CXCR4^+^-CXCR7^+^ complementation cell lines. **Figure D:** Photon flux values for recruitment of β-arrestin 2-CBC to CXCR4-CBRN in 2x β-arrestin 2 and 1x β-arrestin 2 cells corresponding to Fig 6C. **Figure E:** Effect of Changing Receptor Numbers on Beta-arrestin Recruitment to CXCR4 and CXCR7 in Co-expressing Cells. **Figure F:** Expanded Sensitivity Analysis for β-arrestin 2 recruitment to CXCR4 and CXCR7 in Co-expressing Cells. **Table A:** Validation of receptor expression by qRT-PCR. **Table B:** Model Equations. **Table B1:** Equations for cellular events. **Table B2:** CXCR4^+^ Cells. **Table B3:** CXCR7^+^ Cells. **Table B4:** CXCR4^+^-CXCR7^+^ Cells. **Table C:** Cell-surface receptor numbers in CXCR4^+^, CXCR7^+^, and CXCR4^+^-CXCR7^+^ cells. **Table D:** Results of Sensitivity Analysis.
(PDF)
