# Supplementary material for: CXCR7 Controls Competition for Recruitment of β-Arrestin 2 in Cells Expressing Both CXCR4 and CXCR7
Source: PLoS One. 2014 Jun 4;9(6):e98328. doi: 10.1371/journal.pone.0098328 (PMC4045718; doi:10.1371/journal.pone.0098328)
Supplement: File S1 — Contains the following files: Figure A: Photon flux values for (A) CXCR4-CBRN and (B) CXCR7-CBGN corresponding to Fig 3A, 3B. Figure B: Molecular Species Contributing to Beta-arrestin 2 Recruitment. Figure C: Recruitment kinetics of 2x β-arrestin 2, 1x β-arrestin 2 and parental CXCR4+-CXCR7+ complementation cell lines. Figure D: Photon flux values for recruitment of β-arrestin 2-CBC to CXCR4-CBRN in 2x β-arrestin 2 and 1x β-arrestin 2 cells corresponding to Fig 6C. Figure E: Effect of Changing Receptor Numbers on Beta-arrestin Recruitment to CXCR4 and CXCR7 in Co-expressing Cells. Figure F: Expanded Sensitivity Analysis for β-arrestin 2 recruitment to CXCR4 and CXCR7 in Co-expressing Cells. Table A: Validation of receptor expression by qRT-PCR. Table B: Model Equations. Table B1: Equations for cellular events. Table B2: CXCR4+ Cells. Table B3: CXCR7+ Cells. Table B4: CXCR4+-CXCR7+ Cells. Table C: Cell-surface receptor numbers in CXCR4+, CXCR7+, and CXCR4+-CXCR7+ cells. Table D: Results of Sensitivity Analysis. (PDF) [file pone.0098328.s001.pdf]

CXCR7 Controls Competition for Recruitment of β-arrestin 2 in Cells Expressing Both CXCR4 and CXCR7

Nathaniel L. Coggins[1,*], Danielle Trakimas[2,*], S. Laura Chang[2], Anna Ehrlich[1], Paramita Ray[1], Kathryn E. Luker[1], Jennifer J. Linderman[2,3, #], Gary D. Luker[1,3,4, #]

Supplementary Information

Supplementary Information

Supplementary Methods.

Supplementary Figure A: Photon flux values for (A) CXCR4-CBRN and (B) CXCR7-CBGN corresponding to Fig 3A, 3B

Supplementary Figure B: Molecular Species Contributing to β-arrestin 2 Recruitment

Supplementary Figure C: Recruitment kinetics of 2x β-arrestin 2, 1x β-arrestin 2 and parental $CXCR4^{+}$-$CXCR7^{+}$ complementation cell lines

Supplementary Figure D: Photon flux values for recruitment of β-arrestin 2-CBC to CXCR4-CBRN in 2x β-arrestin 2 and 1x β-arrestin 2 cells corresponding to Fig 6C.

Supplementary Figure E: Effect of Changing Receptor Numbers on β-arrestin Recruitment to CXCR4 and CXCR7 in Co-expressing Cells

Supplementary Figure F: Expanded Sensitivity Analysis for β-arrestin 2 recruitment to CXCR4 and CXCR7 in Co-expressing Cells.

Supplementary Table A: Validation of receptor expression by qRT-PCR

Supplementary Table B: Model Equations

- **Table B1:** Equations for cellular events
- **Table B2:** $CXCR4^{+}$ Cells
- **Table B3:** $CXCR7^{+}$ Cells
- **Table B4:** $CXCR4^{+}$-$CXCR7^{+}$ Cells

Supplementary Table C: Cell-surface receptor numbers in $CXCR4^{+}$, $CXCR7^{+}$, and $CXCR4^{+}$-$CXCR7^{+}$ cells

Supplementary Table D: Results of Sensitivity Analysis

Supplementary Methods

qRT-PCR. We isolated RNA using Trizol (Life Technologies) and a RNeasy minikit (Qiagen). cDNA was synthesized using random hexamer primers and Reverse Transcription System (Promega). We measured expression of human CXCR4, CXCR7, and GAPDH by qRT-PCR with gene specific PCR primers as described previously.[1] PCR primers are listed in the table at the end of supplemental methods. For each cell line, we determined amounts of CXCR4 or CXCR7 relative to GAPDH as ΔΔCt values (n = 3 each). After 45 cycles of qRT-PCR, we verified sizes of PCR products by gel electrophoresis. For each RNA sample, no PCR product was produced in negative control samples performed without a reverse transcriptase reaction.

Receptor binding assays. We quantified cell surface CXCR4 and CXCR7 on various cell lines using binding of CXCL12 fused to *Gaussia* luciferase (CXCL12-GL) with minor modifications of our previous protocol.[1] We used parental MDA-MB-231 cells as controls. Briefly, we resuspended 5 x 10^5 cells per condition in 100 μl phenol red free DMEM with 0.2% BSA (Millipore) in microfuge tubes and incubated cells on ice for 30 minutes with increasing concentrations of non-bioluminescent CXCL12. In cells co-expressing CXCR4 and CXCR7 complementation reporters, we added 1 μM AMD3100 or CCX733 (ChemoCentryx) to selectively block CXCR4 or CXCR7, respectively. We added approximately 0.1 ng/mL CXCL12-GL to cells for an additional 30 minutes on ice. We washed cells 3 times with ice-cold PBS before quantifying cell-associated bioluminescence from CXCL12-GL (n = 4). We calculated numbers of CXCL12-GL binding sites per cell for CXCR4 or CXCR7 using GraphPad Prism 5 software. The

calculation subtracts binding of CXCL12-GL to control MDA-MB-231 cells, so a value of 0 denotes binding at background levels.

Plasmids. We used N-terminal and C-terminal fragments of click beetle green and red luciferases (Promega) comprising amino acids 2–413 and 395–542, respectively, for each spectral variant.[2] We designated N-terminal fragments as CBGN and CBRN for click beetle green and red, respectively, which confer spectral characteristics of each luciferase. The common C-terminal fragment, which complements with either N-terminal fragment, is designated CBC. We amplified these fragments by PCR using primers shown below. PCR products were digested with appropriate restriction enzymes and ligated to corresponding sites in our previously described firefly luciferase complementation plasmids for CXCR4, CXCR7, and β-arrestin 2.[3,4] The cloning procedure removed firefly luciferase fragments and replaced them with click beetle fragments.

To enable sorting of transduced cell populations, we modified lentiviral vector FUGW to replace green fluorescent protein (GFP) with either mTagBFP, citrine with a nuclear localization sequence, or FP650.[5–7] We generated the vector for citrine with a nuclear localization sequence by replacing AcGFP with citrine in the nuclear localization vector obtained from Clontech. We amplified click beetle complementation constructs by PCR, which transfers a CMV promoter and complementation reporter to the PacI site of the lentiviral vector. We cloned β-arrestin 2-CBC into the vector with FP650. We inserted CBGN fusions for CXCR4 or CXCR7 into a vector with co-expressed mTagBFP, and CBRN fusions were cloned into a vector with nuclear citrine. Amplified

products for complementation reporters were confirmed by DNA sequencing.

PCR Target	Primer Sequence (forward and reverse)
CXCR4	5’-ACGGACAAGTACAGGCTGCAC-3’
qRT-PCR	5’-CCCAGAAGGGAAGCGTGA-3’
CXCR7	5’-AAGAAGATGGTACGCCGTGTCGTCTGCATCCTG-3’
qRT-PCR	5’-CTCGCTGTGCTTCTCCTGGTCACTGGACGCCGAG-3’
GAPDH	5’-GAAGGTGAAGGTCGGAGT-3’
qRT-PCR	5’-GAAGATGGTGATGGGATTTC-3’
CBGN	5’-ATGCACCGGTCGGCGGTGGCTCATCTGGCGGAGGTGTGAAGCGTGAGAAAAATGTCATC-3’
	5’GCATGCGGCCGCTTAGCCGTCGTCGTCGATGGCC 3’
CBRN	5’-ATGCACCGGTCGGCGGTGGCTCATCTGGCGGAGGTGTAAAGCGTGAGAAAAATGTCATCTATG-3’
	5’-GCATGCGGCCGCTTAGCCGTCGTCGTCGATGGCC-3’
CBC	5’-ATGCACCGGTCGGCGGTGGCTCATCTGGCGGAGGTAGCAAGGGTTATGTCAATAACG-3’
	5’-GCATGCGGCCGCTTAACCGCCGGCCTTCTCCAACAATTGTTT

	C-3’
mTagBFP	5’-CGATTCTAGAGCTACCGGACTCAGATCTCGAG -3’
	5’-GCATTCTAGAGTCGCGGCCGCTTCAATTAAGCTTGTGCCCCAGTTTGC-3’
Citrine-NLS	5’-ATGCGCTAGCGCTACCGGTCGCCACCATGGTGAGCAAGG -3’
	5’-GCATCTCGAGATCTGAGTCCGGACTTGTACAGCTCGTCCATGC-3’
FP650	5’-CGATTCTAGAGCTACCGGACTCAGATCTCGAG -3’
	5’-TGTGGTATGGCTGATTATGATC-3’
Transfer to lentivirus	5’-CGATTCTAGAGCTACCGGACTCAGATCTCGAG -3’
	5’-TGTGGTATGGCTGATTATGATC-3’

Supplementary Figure A. Arbitrary photon flux values for CXCL12-dependent recruitment of β-arrestin 2 to (A) CXCR4-CBRN or (B) CXCR7-CBGN. Data normalized to vehicle control treated cells (0 ng/ml CXCL12) are shown in Fig 3A and 3B, respectively.

A

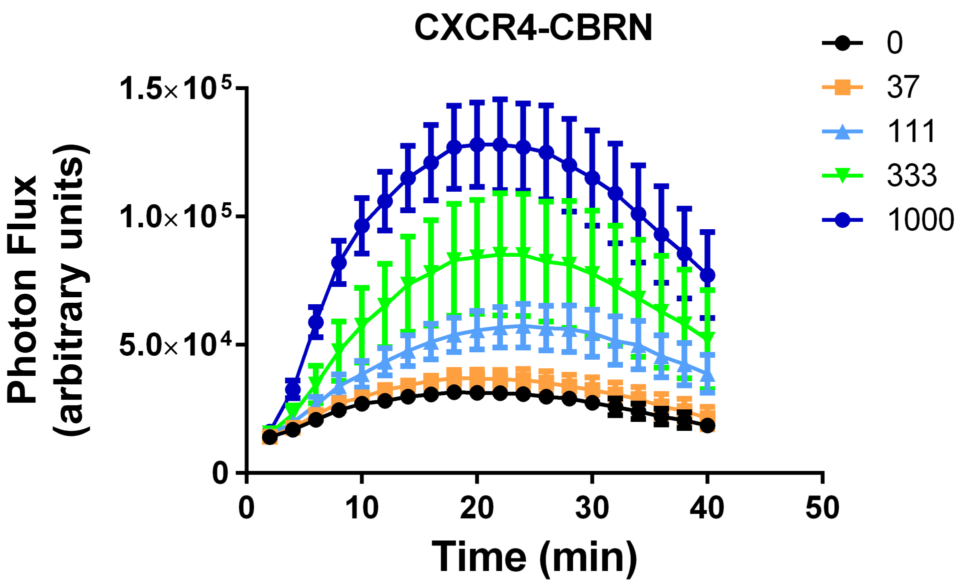

B

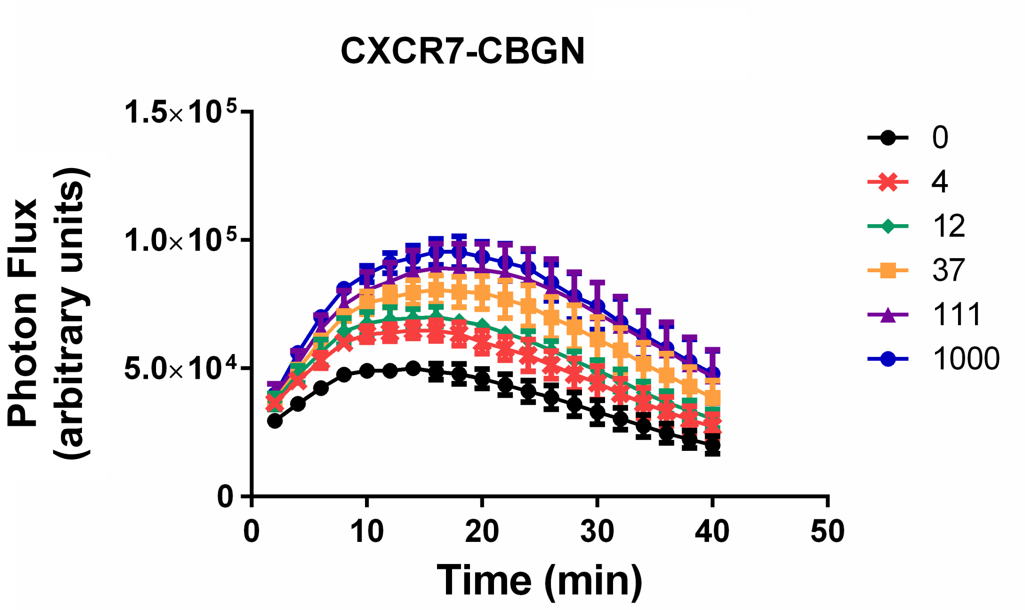

Supplementary Figure B: Molecular Species Contributing to β-arrestin 2 Recruitment Individual species that contribute to β-arrestin 2 recruitment to CXCR4 in $CXCR4^{+}$ cells (A), CXCR7 in $CXCR7^{+}$ cells (B), CXCR4 in $CXCR4^{+}$-$CXCR7^{+}$ cells (C), and CXCR7 in $CXCR4^{+}$-$CXCR7^{+}$ cells (D). For all, CXCL12 = 1000 ng/ml. Species (free receptor bound to probe-labeled arrestin, ligand-bound receptor bound to probe-labeled arrestin, and internalized complexes) are as defined in Table 1.

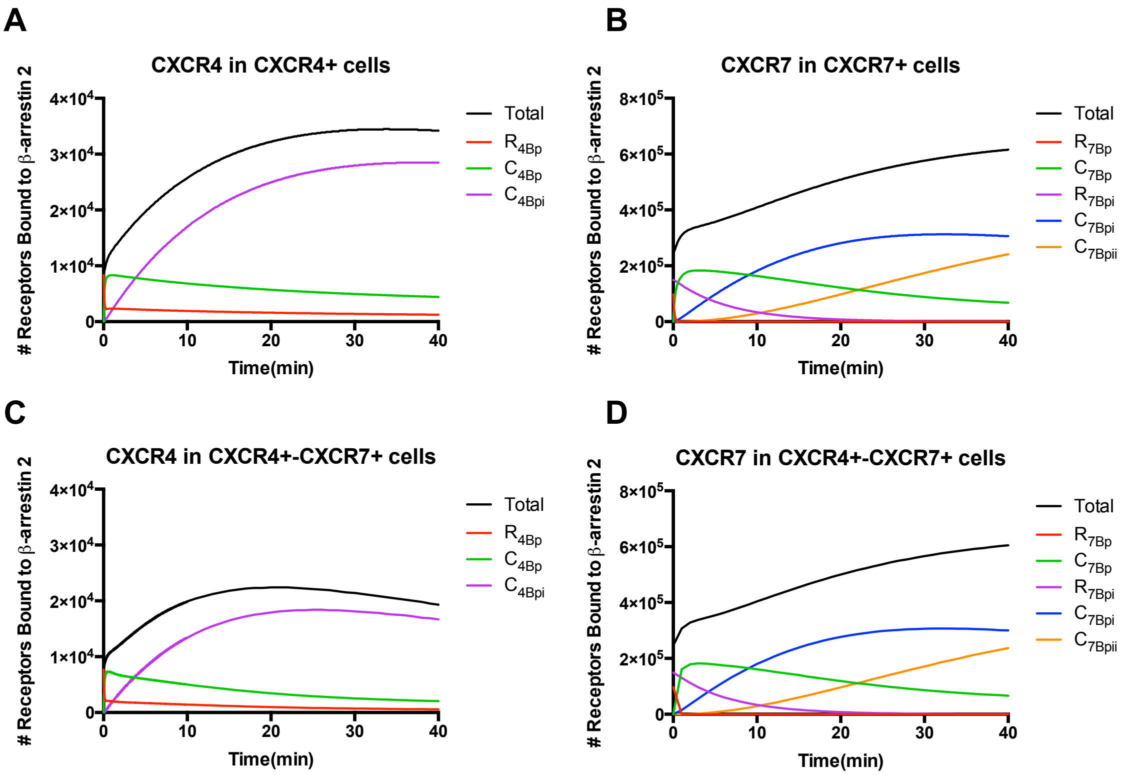

Supplementary Figure C: Recruitment kinetics of 2x β-arrestin 2, 1x β-arrestin 2 and parental CXCR4^{+}-CXCR7^{+} complementation cell lines

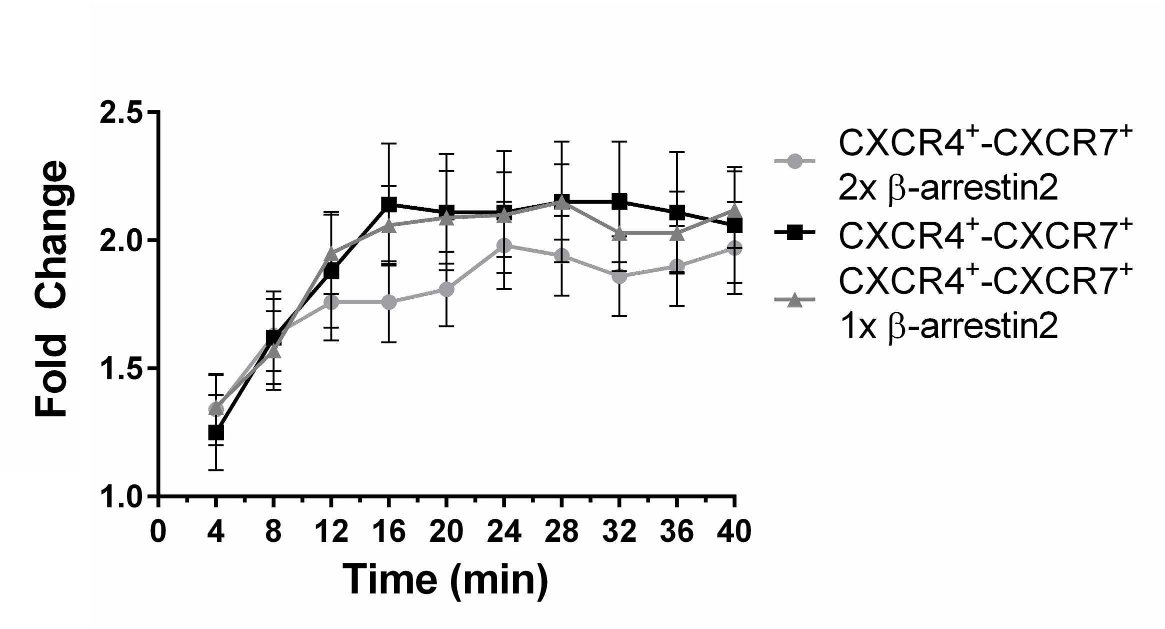

Parental CXCR4^{+}-CXCR7^{+} cells were sorted for high or low expression of FP650, the fluorophore that is co-expressed with β-arrestin 2-CBC. Cells sorted for low FP650 expression (CXCR4^{+}-CXCR7^{+} 1x β-arrestin 2) showed β-arrestin 2 recruitment kinetics that were consistent with the parental CXCR4^{+}-CXCR7^{+} complementation cell line. Therefore, modeling parameters used to describe parental CXCR4^{+}-CXCR7^{+} cells were conserved when generating β-arrestin 2 recruitment outputs for CXCR4^{+}-CXCR7^{+} 1x β-arrestin 2 cells. In contrast, recruitment kinetics of cells that expressed high levels of FP650 (CXCR4^{+}-CXCR7^{+} 2x β-arrestin 2) deviated from recruitment kinetics of parental CXCR4^{+}-CXCR7^{+} and CXCR4^{+}-CXCR7^{+} 1x β-arrestin 2 cell lines.

Supplementary Figure D. Raw photon flux data for extended time course of β-arrestin 2-CBC recruitment to CXCR4-CBRN in CXCR4^{+}-CXCR7^{+} cells expressing 1x or 2x β-arrestin 2-CBC. Graph corresponds with normalized values presented in Fig 6C.

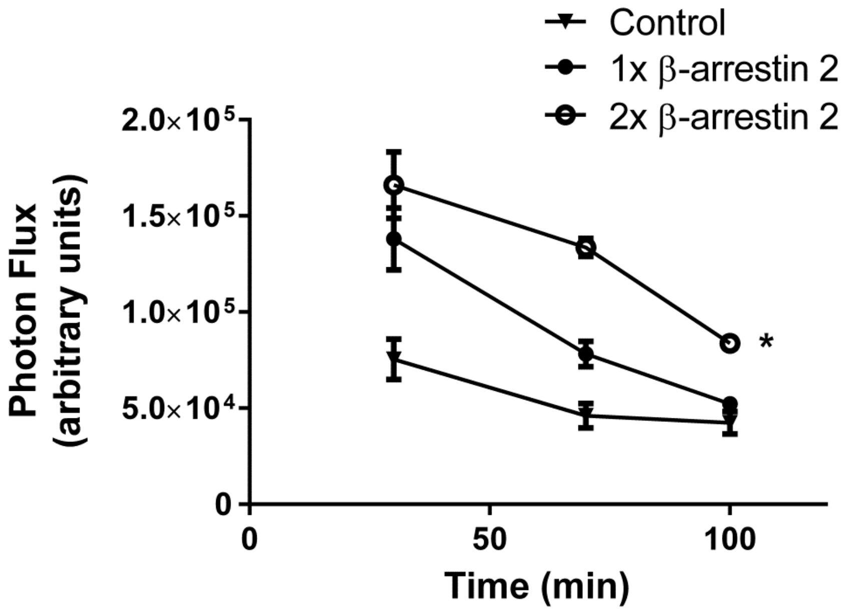

Supplementary Figure E: β-arrestin 2 Recruitment to CXCR4 and CXCR7 in Co-expressing Cells is Affected by Receptor and β-arrestin 2 Levels.

	Molecules per cell			
	**CXCR4**	**CXCR7**	**β-arrestin 2**	**CXCR4:CXCR7**
**1. Base Case**	$6.0x10^5$	$1.5x10^6$	$1.25x10^6$	2:5
**2. Increasing β-arrestin 2**	$6.0x10^5$	$1.5x10^6$	$2.0x10^6$	2:5
**3. Decreasing CXCR7**	$6.0x10^5$	$2.0x10^5$	$1.25x10^6$	3:1
**4. Increasing CXCR7**	$6.0x10^5$	$3.0x10^6$	$1.25x10^6$	1:5
**5. Increasing CXCR4**	$7.5x10^5$	$1.5x10^6$	$1.25x10^6$	5:1

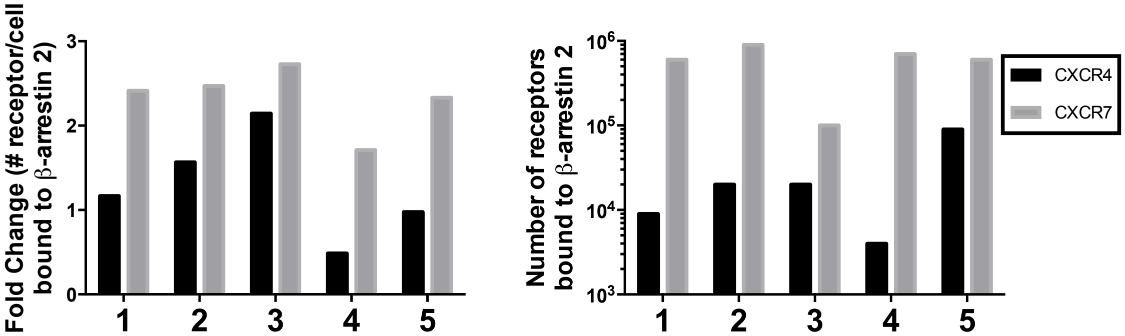

The computational model can be used to examine effects of receptor and β-arrestin 2 levels on CXCR4- and CXCR7- β-arrestin 2 interactions. Values represent β-arrestin 2 recruitment after 40 minutes of treatment with 111 ng/mL CXCL12. Altering β-arrestin 2 or CXCR7 levels affect CXCR4 binding to β-arrestin 2; however, altering levels of CXCR4 only minimally changes CXCR7 interactions with β-arrestin 2.

Supplementary Figure F: Expanded Sensitivity Analysis for β-arrestin 2 recruitment to CXCR4 and CXCR7 in Co-expressing Cells.

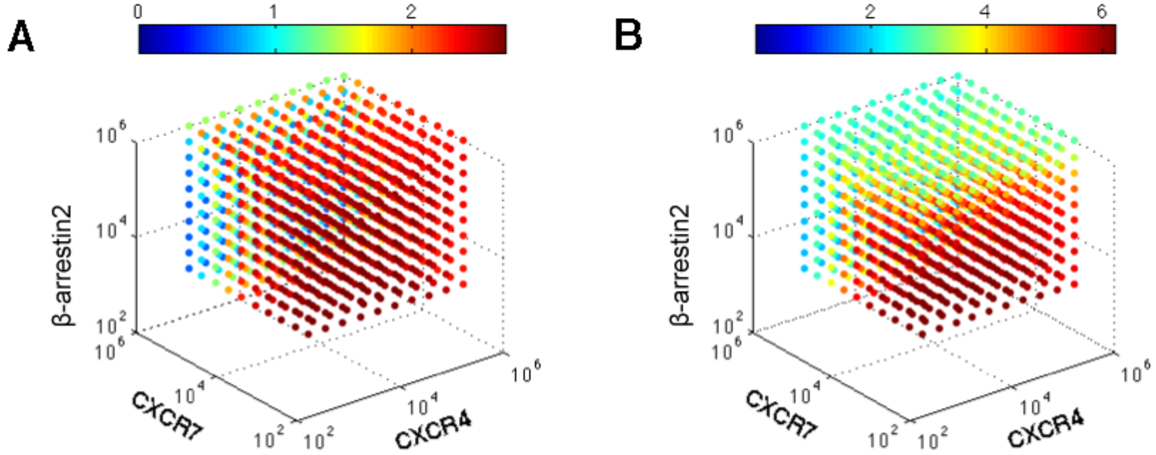

Fold change of β-arrestin 2 recruitment to CXCR4 (A) and CXCR7 (B) after 40 minutes of treatment with 111 ng/mL CXCL12. β-arrestin 2, CXCR4, and CXCR7 were sampled uniformly on a log-scale from 10^3 (physiological range) to 10^6 (overexpression range). Endogenous β-arrestin 2 was set to 0 and all kinetic parameters are as described in Table 2. Colors represent the fold change of β-arrestin 2 to CXCR4 or CXCR7; colorbar limits range from 0 to the maximum calculated fold change value. Consistent with Supplemental Figure 5, altering CXCR7 levels affects CXCR4 binding to β-arrestin 2. However, altering CXCR4 levels has little effect on CXCR7 interactions with β-arrestin 2. In addition, β-arrestin 2 levels greater than 10^5 can affect CXCR7 binding to β-arrestin 2.

Supplementary Table A. Validation of receptor expression by qRT-PCR.

Cell Line	Receptor primers	ΔΔCt ± SEM
**$CXCR4^+$**	hCXCR4	-6.48 ± 0.13
	hCXCR7	0.84 ± 0.13
**$CXCR7^+$**	hCXCR4	0.29 ± 0.67
	hCXCR7	-9.30 ± 0.64
**$CXCR4^+$-$CXCR7^+$**	hCXCR4	-8.76 ± 0.62
	hCXCR7	-10.16 ± 0.94

Each cell line was analyzed for CXCR4 or CXCR7 mRNA by qRT-PCR. ΔΔCt values were calculated by first determining the difference between amplification cycle numbers for the target mRNA and GAPDH (ΔCt) for each cell line. The difference between ΔCt for receptor species in $CXCR4^+$, $CXCR7^+$, or $CXCR4^+$-$CXCR7^+$ cells and ΔCt for the same receptor in 231 control cells was calculated (ΔΔCt) and presented as mean values ± SEM (n = 3). A negative value for ΔΔCt means that mRNA for a receptor was amplified in fewer cycles relative to 231 control cells, therefore indicating more abundant receptor mRNA. A representative gel of qRT-PCR products after 45 cycles is shown below for mRNA extracted and amplified from listed cell lines. Control RNA samples processed without a reverse transcriptase reaction did not produce a product.

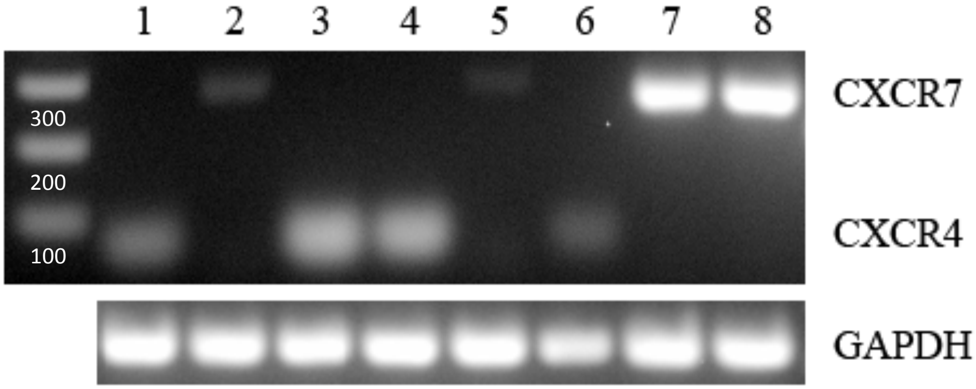

1: Control MDA-MB-231 cells amplified with hCXCR4 primers.

2: MDA-MB-231 $CXCR4^+$ cells amplified with hCXCR7 primers.

3: MDA-MB-231 $CXCR4^+$ cells amplified with hCXCR4 primers.

4: MDA-MB-231 $CXCR4^+$-$CXCR7^+$ cells amplified with hCXCR4 primers.

5: Control MDA-MB-231 cells amplified with hCXCR7 primers.

6: MDA-MB-231 $CXCR7^+$ cells amplified with hCXCR4 primers.

7: MDA-MB-231 $CXCR7^+$ cells amplified with hCXCR7 primers.

8: MDA-MB-231 $CXCR4^+$-$CXCR7^+$ cells amplified with hCXCR7 primers.

Supplementary Table B: Model Equations

Table SB1: Equations for cellular events

**Cellular Event**	**Equation for Event**
**Ligand binding to free receptors**	
L_{12} to R_4	$v_1 = k_{f,L_{12},4}([R_4][L_{12}] - K_{D,R_4,L_{12}}[C_4])$
L_{12} to R_7	$v_2 = k_{f,L_{12},7}([R_7][L_{12}] - K_{D,R_7,L_{12}}[C_7])$
**Ligand binding to receptor-β-arrestin complexes**	
L_{12} to R_{4B_e}	$v_3 = k_{f,L_{12},4}([R_{4B_e}][L_{12}] - K_{D,R_{4B},L_{12}}[C_{4B_e}])$
L_{12} to R_{7B_e}	$v_4 = k_{f,L_{12},7}([R_{7B_e}][L_{12}] - K_{D,R_{7B},L_{12}}[C_{7B_e}])$
L_{12} to R_{4B_p}	$v_5 = k_{f,L_{12},4}([R_{4B_p}][L_{12}] - K_{D,R_{4B},L_{12}}[C_{4B_p}])$
L_{12} to R_{7B_p}	$v_6 = k_{f,L_{12},7}([R_{7B_p}][L_{12}] - K_{D,R_{7B},L_{12}}[C_{7B_p}])$
**β-arrestin binding to free receptors**	
B_e to R_4	$v_7 = k_{f,B,4}([R_4][B_e] - K_{D,R_4,B}[R_{4B_e}])$
B_e to R_7	$v_8 = k_{f,B,7}([R_7][B_e] - K_{D,R_7,B}[R_{7B_e}])$
B_p to R_4	$v_9 = k_{f,B,4}([R_4][B_p] - K_{D,R_4,B}[R_{4B_p}])$
B_p to R_7	$v_{10} = k_{f,B,7}([R_7][B_p] - K_{D,R_7,B}[R_{7B_p}])$
**β-arrestin binding to ligand-bound receptors**	
B_e to C_4	$v_{11} = k_{f,B,4}([C_4][B_e] - K_{D,C_4,B}[C_{4B_e}])$
B_e to C_7	$v_{12} = k_{f,B,7}([C_7][B_e] - K_{D,C_7,B}[C_{7B_e}])$
B_p to C_4	$v_{13} = k_{f,B,4}([C_4][B_p] - K_{D,C_4,B}[C_{4B_p}])$
B_p to C_7	$v_{14} = k_{f,B,7}([C_7][B_p] - K_{D,C_7,B}[C_{7B_p}])$
**Internalization of cell surface receptor-β-arrestin complexes**	
Internalization of R_{4B_e}	$v_{15} = k_{e,R_{4B}}[R_{4B_e}]$
Internalization of R_{7B_e}	$v_{16} = k_{e,R_{7B}}[R_{7B_e}]$
Internalization of R_{4B_p}	$v_{17} = k_{e,R_{4B}}[R_{4B_p}]$
Internalization of R_{7B_p}	$v_{18} = k_{e,R_{7B}}[R_{7B_p}]$
Internalization of C_{4B_e}	$v_{19} = k_{e,C_{4B}}[C_{4B_e}]$
Internalization of C_{7B_e}	$v_{20} = k_{e,C_{7B}}[C_{7B_e}]$
Internalization of C_{4B_p}	$v_{21} = k_{e,C_{4B}}[C_{4B_p}]$
Internalization of C_{7B_p}	$v_{22} = k_{e,C_{7B}}[C_{7B_p}]$
**Dissociation of β-arrestin from internalized receptor-β-arrestin complexes**	
B_e from $R_{7B_{e}i}$	$v_{23} = k_{off,B,7}[R_{7B_{e}i}]$
B_p from $R_{7B_{p}i}$	$v_{24} = k_{off,B,7}[R_{7B_{p}i}]$
B_e from $C_{4B_{e}i}$	$v_{25} = k_{off,B,4}[C_{4B_{e}i}]$
B_p from $C_{4B_{p}i}$	$v_{26} = k_{off,B,4}[C_{4B_{p}i}]$
**Trafficking of internalized receptor-β-arrestin complexes to late endosomes**	
Trafficking of $C_{7B_{e}i}$ to late endosomes	$v_{27} = k_{e,C_{7Bi}}[C_{7B_{e}i}]$
Trafficking of $C_{7B_{p}i}$ to late endosomes	$v_{28} = k_{e,C_{7Bi}}[C_{7B_{p}i}]$
**Recycling of internalized receptors**	

Recycling of $R_{4B_{e}i}$	$\upsilon_{29} = k_{rec,R_{4Bi}}\left[R_{4B_{e}i}\right]$
Recycling of $R_{7B_{e}ii}$	$\upsilon_{30} = k_{rec,R_{7Bii}}\left[R_{7B_{e}ii}\right]$
Recycling of $R_{4B_{p}i}$	$\upsilon_{31} = k_{rec,R_{4Bi}}\left[R_{4B_{p}i}\right]$
Recycling of $R_{7B_{p}ii}$	$\upsilon_{32} = k_{rec,R_{7Bii}}\left[R_{7B_{p}ii}\right]$
Recycling of $C_{7B_{e}ii}$	$\upsilon_{33} = k_{rec,C_{7Bii}}\left[C_{7B_{e}ii}\right]$
Recycling of $C_{7B_{p}ii}$	$\upsilon_{34} = k_{rec,C_{7Bii}}\left[C_{7B_{p}ii}\right]$
**Degradation of internalized receptors and ligand**	
Degradation of $C_{4B_{e}ii}$	$\upsilon_{35} = k_{deg,C_{4Bii}}\left[C_{4B_{e}ii}\right]$
Degradation of $C_{4B_{p}ii}$	$\upsilon_{36} = k_{deg,C_{4Bii}}\left[C_{4B_{p}ii}\right]$
Degradation of L_{12i}	$\upsilon_{37} = k_{deg,L_{12i}}\left[L_{12i}\right]$

Table B2: $CXCR4^{+}$ Cells

Differential equations describing the change in the concentration of species in $CXCR4^{+}$ cells over time.

Equations (#/cell/s)
$\frac{d[R_4]}{dt} = -v_1 - v_7 - v_9 + v_{29} + v_{31}$
$\frac{d[R_{4B_e}]}{dt} = +v_7 - v_3 - v_{15}$
$\frac{d[R_{4B_p}]}{dt} = +v_9 - v_5 - v_{17}$
$\frac{d[C_4]}{dt} = +v_1 - v_{11} - v_{13}$
$\frac{d[C_{4B_e}]}{dt} = +v_3 + v_{11} - v_{19}$
$\frac{d[C_{4B_p}]}{dt} = +v_5 + v_{13} - v_{21}$
$\frac{d[R_{4B_e i}]}{dt} = +v_{15} - v_{29}$
$\frac{d[R_{4B_p i}]}{dt} = +v_{17} - v_{31}$
$\frac{d[C_{4B_e i}]}{dt} = +v_{19} - v_{25}$
$\frac{d[C_{4B_p i}]}{dt} = +v_{21} - v_{26}$
$\frac{d[C_{4B_e ii}]}{dt} = +v_{25} - v_{35}$
$\frac{d[C_{4B_p ii}]}{dt} = +v_{26} - v_{36}$
$*\frac{d[L_{12}]}{dt} = (-v_1 - v_3 - v_5)\times\frac{n_4\times10^9}{V\times N_{Av}}$
$\frac{d[L_{12i}]}{dt} = +v_{19} + v_{21} - v_{37}$
$\frac{d[B_e]}{dt} = -v_7 - v_{11} + v_{15} + v_{25}$

$\frac{d[B_p]}{dt} = -v_9 - v_{13} + v_{17} + v_{26}$

*Units for this equation are (nM/s)

N_{Av} : Avogadro's number

Table B3: CXCR7$^+$ Cells
Differential equations describing the change in the concentration of species in CXCR7$^+$ cells over time.

**Equations (#/cell/s)**
$\frac{d[R_7]}{dt} = -v_2 - v_8 - v_{10} + v_{30} + v_{32} + v_{33} + v_{34}$
$\frac{d[R_{7B_e}]}{dt} = +v_8 - v_4 - v_{16}$
$\frac{d[R_{7B_p}]}{dt} = +v_{10} - v_6 - v_{18}$
$\frac{d[C_7]}{dt} = +v_2 - v_{12} - v_{14}$
$\frac{d[C_{7B_e}]}{dt} = +v_4 + v_{12} - v_{20}$
$\frac{d[C_{7B_p}]}{dt} = +v_6 + v_{14} - v_{22}$
$\frac{d[R_{7B_ei}]}{dt} = +v_{16} - v_{23}$
$\frac{d[R_{7B_pi}]}{dt} = +v_{18} - v_{24}$
$\frac{d[C_{7B_ei}]}{dt} = +v_{20} - v_{27}$
$\frac{d[C_{7B_pi}]}{dt} = +v_{22} - v_{28}$
$\frac{d[R_{7B_eii}]}{dt} = +v_{23} - v_{30}$
$\frac{d[R_{7B_pii}]}{dt} = +v_{24} - v_{32}$
$\frac{d[C_{7B_eii}]}{dt} = +v_{27} - v_{33}$
$\frac{d[C_{7B_pii}]}{dt} = +v_{28} - v_{34}$
$* \frac{d[L_{12}]}{dt} = (-v_2 - v_4 - v_6) \times \frac{n_7 \times 10^9}{V \times N_{Av}}$
$\frac{d[L_{12i}]}{dt} = +v_{20} + v_{22} - v_{37}$
$\frac{d[B_e]}{dt} = -v_8 - v_{12} + v_{23} + v_{33}$
$\frac{d[B_p]}{dt} = -v_{10} - v_{14} + v_{24} + v_{34}$

*Units for this equation are (nM/s)

N_{Av} : Avogadro's number

Table B4: CXCR4^{+}-CXCR7^{+} Cells
Differential equations describing the change in the concentration of species in CXCR4^{+}-CXCR7^{+} cells over time.

All equations from Tables S4B and S4C apply, except for the following:

Equations (#/cell/s)
$* \frac{d[L_{12}]}{dt} = (-v_1 - v_3 - v_5 - v_2 - v_4 - v_6) \times \frac{n_{47} \times 10^9}{V \times N_{Av}}$
$\frac{d[L_{12i}]}{dt} = +v_{19} + v_{21} + v_{20} + v_{22} - v_{37}$
$\frac{d[B_e]}{dt} = -v_7 - v_{11} + v_{15} + v_{25} - v_8 - v_{12} + v_{23} + v_{33}$
$\frac{d[B_p]}{dt} = -v_9 - v_{13} + v_{17} + v_{26} - v_{10} - v_{14} + v_{24} + v_{34}$

*Units for this equation are (nM/s)

N_{Av} : Avogadro's number

Supplementary Table C: Cell-surface receptor numbers in $CXCR4^+$, $CXCR7^+$, and $CXCR4^+$-$CXCR7^+$ cells

**Cell Type**	**CXCR4 (#/cell)**	**CXCR7 (#/cell)**
**$CXCR4^+$**	2.3×10^5	0
**$CXCR7^+$**	0	9.1×10^5
**$CXCR4^+$-$CXCR7^+$**	2.2×10^5	2.9×10^5

Supplementary Table D: Sensitivity Analysis

**Parameter**	**2 min**	**10 min**	**20 min**	**90 min**
$k_{f,L_{12},4}$	++	n.s.	n.s.	n.s.
$k_{f,L_{12},7}$	n.s.	+	++	+
$k_{f,B,4}$	+	+	n.s.	n.s.
$k_{f,B,7}$	n.s.	n.s.	n.s.	-
$K_{D,R_4,L_{12}}$	--	---	---	---
$K_{D,R_7,L_{12}}$	n.s.	n.s.	-	--
$K_{D,R_4,B}$	+++	+++	+++	+++
$K_{D,R_7,B}$	+++	+++	+++	+++
$K_{D,C_4,B}$	---	---	---	---
$K_{D,C_7,B}$	---	---	---	---
$k_{e,R_{4B}}$	++	+++	+++	+++
$k_{e,R_{7B}}$	n.s.	--	---	---
$k_{e,C_{4B}}$	++	+++	+++	+
$k_{e,C_{7B}}$	+	+++	+++	+++
$k_{off,B,4}$	n.s.	---	---	---
$k_{off,B,7}$	n.s.	+++	+++	+++
$k_{e,C_{7Bi}}$	n.s.	n.s.	n.s.	-
$k_{rec,R_{4Bi}}$	n.s.	n.s.	n.s.	--
$k_{rec,R_{7Bii}}$	n.s.	n.s.	--	---
$k_{rec,C_{7Bii}}$	n.s.	n.s.	n.s.	---
$k_{deg,C_{4Bii}}$	n.s.	n.s.	n.s.	n.s.
$k_{deg,L_{12i}}$	n.s.	n.s.	n.s.	n.s.

Sensitivity analysis was performed using 111 ng/ml CXCL12; analyses for other ligand concentrations are similar and are not shown. Each parameter was varied an order of magnitude above and below its model value, using a log scale. The output of interest was fold-change of β-arrestin bound to either CXCR4 or CXCR7 in single-expressing cells at 2, 10, 20 and 90 minutes after treatment with CXCL12. Only parameters with PRCC values > 0.09 or < -0.09 and p-values less than 0.01 were counted as significant. +/- signs represent positive/negative correlations and n.s. indicates a non-significant correlation. The number of signs represents the significance of the correlation:

+/- : $10^{-5} < p < 10^{-2}$

++/-- : $10^{-12} < p < 10^{-5}$

+++/--- : $p < 10^{-12}$

References

1. Luker KE, Mihalko LA, Schmidt BT, Lewin SA, Ray P, et al. (2011) In vivo imaging of ligand receptor binding with Gaussia luciferase complementation. Nat Med 18: 172–177. doi:10.1038/nm.2590; 10.1038/nm.2590.

2. Villalobos V, Naik S, Bruinsma M, Dothager RS, Pan MH, et al. (2010) Dual-color click beetle luciferase heteroprotein fragment complementation assays. Chem Biol 17: 1018–1029. doi:10.1016/j.chembiol.2010.06.018; 10.1016/j.chembiol.2010.06.018.

3. Luker KE, Gupta M, Luker GD (2008) Imaging CXCR4 signaling with firefly luciferase complementation. Anal Chem 80: 5565–5573. doi:10.1021/ac8005457.

4. Luker KE, Gupta M, Steele JM, Foerster BR, Luker GD (2009) Imaging ligand-dependent activation of CXCR7. Neoplasia 11: 1022–1035.

5. Lois C, Hong EJ, Pease S, Brown EJ, Baltimore D (2002) Germline transmission and tissue-specific expression of transgenes delivered by lentiviral vectors. Science 295: 868–872. doi:10.1126/science.1067081.

6. Shcherbo D, Shemiakina II, Ryabova A V, Luker KE, Schmidt BT, et al. (2010) Near-infrared fluorescent proteins. Nat Methods 7: 827–829. doi:10.1038/nmeth.1501; 10.1038/nmeth.1501.

7. Subach OM, Gundorov IS, Yoshimura M, Subach F V, Zhang J, et al. (2008) Conversion of red fluorescent protein into a bright blue probe. Chem Biol 15: 1116–1124. doi:10.1016/j.chembiol.2008.08.006; 10.1016/j.chembiol.2008.08.006.
